# Longitudinal Study Reveals Long-Term Proinflammatory Proteomic Signature After Ischemic Stroke Across Subtypes

**DOI:** 10.1161/STROKEAHA.121.038349

**Published:** 2022-06-10

**Authors:** Tara M. Stanne, Annelie Angerfors, Björn Andersson, Cecilia Brännmark, Lukas Holmegaard, Christina Jern

**Affiliations:** Institute of Biomedicine, Department of Laboratory Medicine, the Sahlgrenska Academy, University of Gothenburg, Sweden (T.M.S., A.A., C.B., C.J.).; Bioinformatics Core Facility, University of Gothenburg, Sweden (B.A.).; Institute of Neuroscience and Physiology, Department of Clinical Neuroscience, the Sahlgrenska Academy, University of Gothenburg, Sweden (L.H.).; Department of Neurology (L.H.), Sahlgrenska University Hospital, Region Västra Götaland, Gothenburg, Sweden.; Department of Clinical Genetics and Genomics (C.J.), Sahlgrenska University Hospital, Region Västra Götaland, Gothenburg, Sweden.

**Keywords:** chemokines, cryptogenic stroke, inflammation, interleukin, ischemic stroke, proteomics

## Abstract

Inflammation contributes both to the pathogenesis of stroke and the response to brain injury. We aimed to identify proteins reflecting the acute-phase response and proteins more likely to reflect proinflammatory processes present before stroke by broadly profiling inflammation-related plasma proteins in a longitudinal ischemic stroke study.

**Methods:**

Participants were from a Swedish ischemic stroke cohort (SAHLSIS [Sahlgrenska Academy Study on Ischemic Stroke], n=600 cases and n=600 controls). Plasma levels of 65 proteins including chemokines, interleukins, surface molecules, and immune receptors were measured once in controls and at 3× in cases: during the acute phase, after 3 months, and for a subgroup (n=223) at 7-year follow-up. Associations between proteins and ischemic stroke or subtype were investigated in multivariable binary regression models corrected for age, sex, vascular risk factors, and multiple testing.

**Results:**

In the acute phase, 48 proteins were significantly and independently associated with ischemic stroke (false discovery rate adjusted *P*<0.05). At 3-month follow-up, 51 proteins and at 7-year follow-up 50 proteins were associated with ischemic stroke. The majority of proteins were upregulated in cases compared with controls (n=34 at all time points) and the most upregulated were CXCL5 (CXC chemokine ligand 5) and OSM (oncostatin M). Generally, large artery and cardioembolic stroke had the highest protein levels. However, several interesting subtype-specific differences were also detected at each time point.

**Conclusions:**

We found inflammation-related proteins that were differentially regulated in ischemic stroke cases compared with controls only in the acute phase and others that remained elevated also at later time points. This latter group of proteins could reflect underlying pathophysiological processes of relevance. Future studies both in terms of disease risk and prognostication are warranted.

Inflammation has an important role in the pathogenesis of ischemic stroke as well as in the acute response to a cerebral ischemic event.^1^ It has long been known that ischemia prompts a robust inflammatory response locally at the site of the brain infarction. More recently, evidence from human and animal models of stroke indicate that global brain inflammation (ie, in areas remote from the injury site) also occurs.^2^ This inflammatory response is complex, involving resident brain cells (eg, microglia and astrocytes), as well as infiltrating peripheral immune cells (eg, neutrophils, T cells, dendritic cells, and macrophages) due to blood-brain barrier disruption.^1^ Together these cells secrete a range of proinflammatory cytokines and chemokines and lead to a peripheral inflammatory reaction.^1^

With regards to this peripheral inflammatory response, circulating levels of both CRP (C-reactive protein) and IL (interleukin)-6 are well established to be elevated during the acute phase of ischemic stroke across etiologic subtypes.^1,3,4^ However, circulating levels of upstream inflammation-related proteins that might be more directly coupled to the underlying mechanisms have, for the most part, not been well-studied in large clinical studies of ischemic stroke.^1,5^ Furthermore, the extent to which the inflammatory state remains during the convalescent phase, and even more long-term after ischemic stroke, is not well explored.

Inflammation also affects different pathological processes that contribute to stroke risk, such as atherosclerosis, small vessel disease, atrial fibrillation, and prothrombotic states.^1,6,7^ It follows that inflammation has previously been implicated in the main etiological subtypes of ischemic stroke: large artery atherosclerosis (LAA), cardioembolic stroke, small artery occlusion stroke (SAO), and cryptogenic stroke.^1,8,9^ Moreover, vessel wall inflammation has been proposed as one of the causes of cervical arterial dissection (CeAD).^10^ Although inflammation appears to be relevant for different ischemic stroke subtypes, few studies on circulating inflammatory plasma proteins have performed subtype-specific analyses.

Here, we aimed to address these knowledge gaps by broadly investigating plasma levels of inflammation-related proteins (including CC and CXC chemokines, interleukins, surface molecules, and immune receptors) at 3 time points in a longitudinal ischemic stroke cohort study and by also performing subtype-stratified analyses. We hypothesized that some proteins would be elevated only in the acute phase and primarily reflect the response to cerebral ischemia per se, whereas others would be elevated also at later time points. This latter group of proteins could potentially reflect underlying pathophysiological processes of relevance and may be subtype-specific.

## Methods

The data that support the findings of this study are available from the corresponding author upon reasonable request. Our study fulfills the STROBE (Strengthening the Reporting of Observational Studies in Epidemiology) checklist guidelines (see Supplemental Material).

### Study Population and Blood Sampling

The present study included 600 cases and 600 controls from the prospective observational study the Sahlgrenska Academy Study on Ischemic Stroke (SAHLSIS), which is described in detail elsewhere.^11^ In brief, cases aged 18 to 69 years presenting with first-ever or recurrent acute ischemic stroke were consecutively recruited at 4 stroke units in Western Sweden between 1998 and 2003. The age group was chosen to reduce potential confounding factors related to comorbidities and the proportion of cases classified as stroke of undetermined cause. All cases underwent computed tomography and 63% underwent magnetic resonance imaging of the brain. Stroke severity was scored as maximum severity within the first 7 days after hospital admission using the Scandinavian Stroke Scale and then converted using an algorithm to the more commonly used National Institutes of Health Stroke Scale (NIHSS).^12^ The inclusion criteria were acute onset of clinical symptoms suggestive of stroke lasting >24 hours and computed tomography scan and magnetic resonance imaging of the brain. Patients were excluded if they showed a cause other than ischemic stroke following evaluation, or had a diagnosis of cancer of advanced stage, infectious hepatitis, or HIV. Community controls were randomly selected from the general population to match the cases with regard to age (±1 year), sex, and geographic residence area between 1999 and 2004. Controls were excluded if they had a history of stroke, coronary heart disease, or peripheral artery disease.

Standardized blood sampling (at 8:30 am–10:30 am after overnight fasting) was performed once in controls and within 10 days (median 4 days, interquartile range, 3–6) of the index stroke event and at 3-month follow-up in cases. For a subgroup of 223 cases, additional blood sampling was performed at a 7-year follow-up. Venous blood was collected in tubes containing 10% by volume EDTA (Vacuette, Greiner Bio-One, Essen, Germany). Plasma was isolated within 2 hours by centrifugation 2000×*g* at 4 °C for 20 minutes, aliquoted, and stored at −80 °C pending analysis. Further details on sample collection dates for cases and controls, as well as missing data can be found in Table S1.

Written informed consent was obtained from all participants or their next-of-kin before enrollment. The study was approved by the Regional Ethics Review Board in Gothenburg (469-99, T553-03, 413-04, T665-07).

### Etiologic Subtypes, Definition of Vascular Risk Factors, and Recurrent Events

Etiologic subtypes of ischemic stroke were classified according to the TOAST (Trial of ORG 10172 in Acute Stroke Treatment) criteria^13^ with minor modifications as described^14^ into the categories LAA (n=73), SAO (n=124), cardioembolic (n=98), cryptogenic stroke (n=162; defined here as no identified cause despite a complete evaluation), CeAD (n=32), other determined cause (n=19), and undetermined stroke (n=92, defined either as incomplete evaluation, n=67 or >1 identified cause, n=25).

Information regarding vascular risk factors was registered at inclusion for controls and at the 3-month and 7-year follow-up for cases by examinations and a structured questionnaire, as described.^11^ Acute phase serum levels of hsCRP (high-sensitivity CRP) were determined as previously described,^3^ and peripheral white blood cell count and neutrophil-lymphocyte ratios were determined at the Department of Clinical Chemistry at our hospital as a part of the clinical routine. Further details can be found in the Supplemental Material.

A stroke neurologist identified recurrent strokes and coronary events (defined as a myocardial infarction, hospitalization for unstable angina, acute coronary artery bypass grafting, or percutaneous coronary intervention) before the 3-month follow-up by medical history and review of the medical records. To obtain data on recurrent strokes and coronary events during long-term follow-up, the Swedish National Hospital Discharge Registry was used and events were confirmed by reviewing the corresponding medical record.^15^

### Measurement of Inflammation-Related Protein Biomarkers

Plasma levels of inflammation-related proteins were analyzed using a proximity extension assay as described in detail elsewhere and in the Supplemental Material.^16^ This commercial multiplex panel targets 92 proteins with documented or suggested involvement in inflammatory processes or disease (Proseek Multiplex Inflammation I, Olink Bioscience, Uppsala, Sweden). A list of these proteins with corresponding UniProt Identities are provided in Table S2. Of note, many of these proteins have alternative protein names and here we use the nomenclature adapted by Olink. Normalization of data was performed in GenEx software using Olink Wizard providing Normalized Protein eXpression data on a log2-scale where 1 unit higher Normalized Protein eXpression value represents a doubling of the measured protein concentration.^16^ All analyses were performed by a board-certified laboratory technician blinded to the clinical information. The samples from this study were analyzed on 2 occasions using 2 batches of assays: the first was a pilot and included 200 cases with a complete set of 4 samples (ie, acute, 3-month, 7-year, and matched controls); the remaining samples were run 6 months later. To minimize the impact of inter-run variability or batch effects, all samples from each case were placed on the same plate as the corresponding matched control. Furthermore, TOAST subtypes were randomly distributed among the plates in both runs.

### Missing Data

Plasma samples were missing for 20 controls, 39 cases in the acute phase, 51 cases at 3-month, and 0 cases at 7-year follow-up (Table S2). Samples that failed the Olink technical quality controls were excluded (controls, n=6; acute phase, n=5; 3 months, n=2; and 7 years, n=0). The total number of samples included was therefore controls, n=574; acute phase, n=556; 3 months, n=547; and 7 years, n=223.

#### Statistical Analyses

Of the 92 proteins, 65 showed a prespecified call rate >80% and were included in analyses (for details see Table S1). Notable examples of excluded proteins include IL-1, IL-2, IL-4, and TNF (tumor necrosis factor)-α. For each protein, data below the limit of detection were replaced by limit of detection/2. Data were visually inspected in terms of clustering and outliers using principal component analysis. No evidence of systematic errors due to date or site of sample collection was found (Figure S1). To remove unwanted variation due to batch effects, ComBat adjustment was used (package sva). Baseline characteristics were compared with χ^2^ test for proportions and Student *t* test. Correlations between proteins (ie, Normalized Protein eXpression values) and clinical variables, age, hsCRP, peripheral blood cell counts, and day of blood draw were assessed by Pearson correlation coefficients. Univariable binary logistic regression was used to analyze associations between individual proteins and ischemic stroke or stroke subtype. Prespecified adjustments for age, sex, and vascular risk factors (hypertension, smoking, body mass index, and diabetes) were made in multivariable models. As the Normalized Protein eXpression value is given on a log2 scale, the yielded odds ratio corresponds to the predicted increased odds of stroke per each doubling of the protein levels. *P*-values were adjusted for multiple testing by false discovery rate (adjusted *P* or *q*<0.05).

The following sensitivity analyses were also conducted: (1) Reproducibility: The study population was randomly split into 2 samples. TOAST subtype was used as a grouping variable to assure equal distribution in the 2 samples. (2) Signs of infection: Medical journal records for cases with acute levels of hsCRP >50 mg/L or white blood cell count >10×10^9^/L or neutrophil-lymphocyte ratios >5 were reviewed. Eighteen cases were confirmed to exhibit clinical signs of infection and were excluded from a sensitivity analysis. (3) Recurrent vascular events: For 3-month and 7-year protein levels, sensitivity analyses were performed to exclude cases with a recurrent stroke or coronary event before follow-up (3 months, n=32; 7 years, n=47). (4) 7-year age-matched controls: regressions were performed for 7-year protein levels using a subset of controls matched for sex and age of cases at the 7-year follow-up. (5) Patients that attended 7-year follow-up: A subanalysis was also performed for acute and 3-month proteins only in the subset of patients that attended the 7-year follow-up. Statistical analyses were performed in IBM SPSS Statistics 26 or R version 4.1.0, and all tests were 2-tailed. The packages ggplot2 and corrplot were used for visualization.

## Results

### Study Population

Baseline characteristics of cases and controls in SAHLSIS and vascular risk factors at 7-year follow-up are summarized in the Table. A higher percentage of cases that participated in the long-term follow-up had an index cryptogenic stroke, whereas a lower percentage had a cardioembolic stroke, due to a higher mortality rate in the latter group.

### Correlations

Many proteins exhibited weak (Pearson *r*, 0.25–0.5) or moderate (*r*, 0.5–0.75) correlations with each other in both cases and controls and a few were strongly correlated (*r*  >0.75; Figure S2). The majority of proteins were negligibly (Pearson *r*  <0.25) correlated with age, body mass index, white blood cell count, and time of blood draw. Previously measured hsCRP^3^ was significantly and directly correlated to 4 proteins in controls and 15 in cases, with strongest correlation to IL-6 in both (*r*  =0.49 in controls and *r*  =0.66 in cases) and inversely correlated with 4 proteins in cases. Neutrophil-lymphocyte ratio was not correlated to any protein in controls; however, in cases hsCRP^3^ (*r =*0.40) and 5 other proteins were directly correlated to neutrophil-lymphocyte ratio (strongest IL-6, *r*  =0.45), and 6 were inversely correlated. The protein with the strongest direct correlation to stroke severity (NIHSS) was previously measured hsCRP^3^ (*r*  =0.42) followed by IL-6 (*r*  =0.38), EN-RAGE [extracellular newly identified receptor for advanced glycation end-products binding protein; alternative name S100 calcium-binding protein A12] (*r  *=0.32), CCL23 (*r*  =0.29), OSM (oncostatin M; *r*  =0.28), and 3 proteins were inversely correlated (Flt3L [FMS-like tyrosine kinase 3 ligand], *r*  =−0.25; TRAIL [TNF-related apoptosis-inducing ligand; alternative name TNFSF10, TNF superfamily member 10], *r*  =−0.28; TRANCE [TNF-related activation-induced cytokine; alternative name TNFSF11], *r  =*−0.29; for details see Table S3).

### Majority of Inflammatory-Related Proteins Were Higher in Ischemic Stroke Cases Compared With Controls, Across Time Points

**Figure 1.**
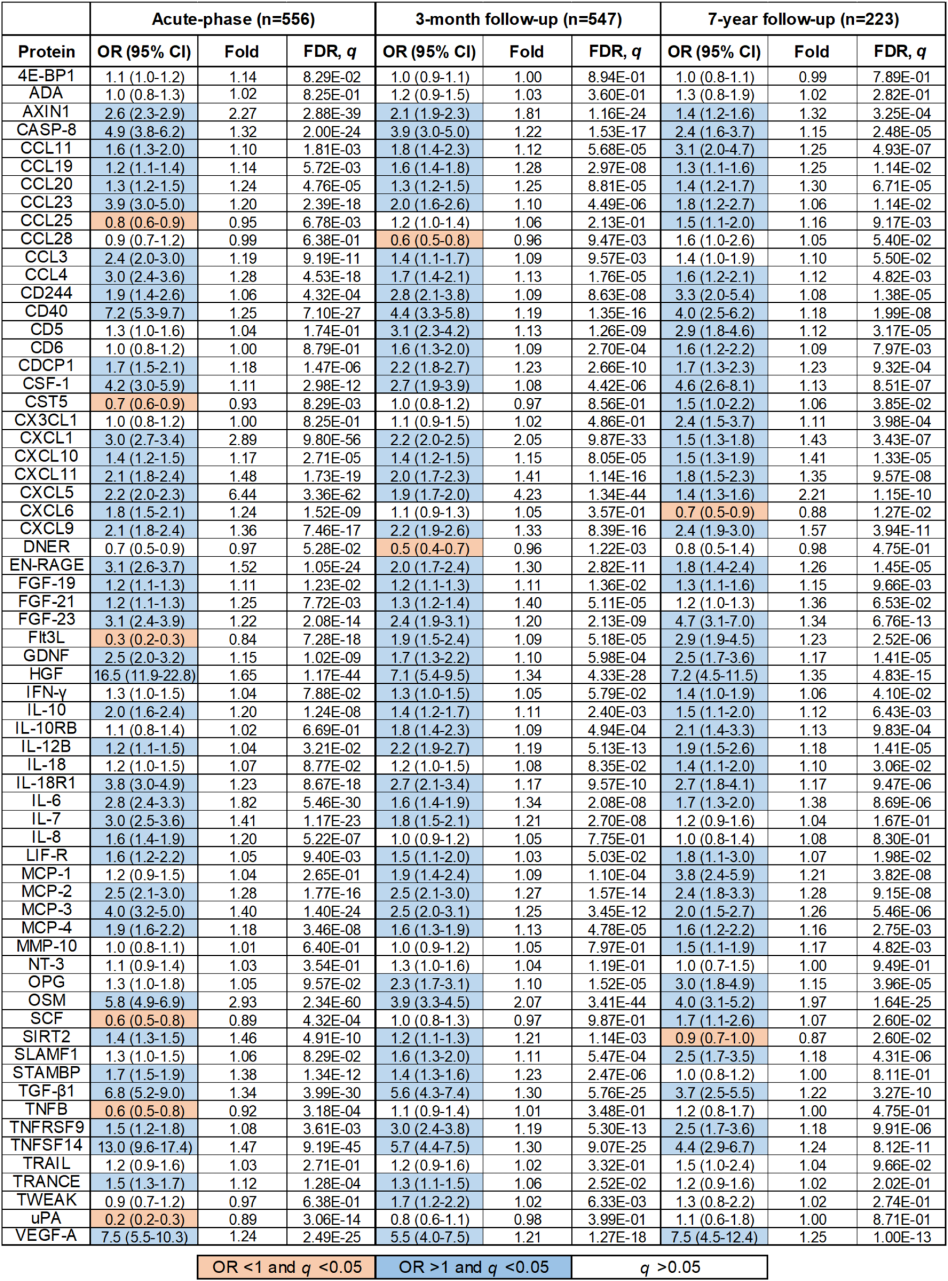
**Odds ratios (ORs) and 95% CI for ischemic stroke per one Normalized Protein eXpression value (ie, doubling of protein concentration) as compared to controls during the acute-phase, 3-mo, and 7-y follow-up.** Binary regressions were adjusted for age, sex, body mass index, diabetes, hypertension, smoking, and multiple testing (false discovery rate [FDR]). The mean fold-change between cases and controls are also indicated. White, nonsignificant (*q* >0.05); blue, significant (*q* <0.05) and elevated in cases; red, significant (*q*<0.05) and lower in cases compared to controls. A complete list of all protein names with corresponding UniProt identities are provided in Table S2. CCL indicates CC chemokine ligand; CXCL, CXC chemokine ligand; FGF, fibroblast growth factor; Flt3L, FMS-like tyrosine kinase 3 ligand; HGF, hepatocyte growth factor; IL, interleukin; INF, interferon; MCP, monocyte chemoattractant protein; OSM, oncostatin M; SIRT, sirtuin; TGF, transforming growth factor; TNF, tumor necrosis factor; TRAIL, TNF-related apoptosis-inducing ligand; and TRANCE, TNF-related activation-induced cytokine.

Results from the multivariable binary regression analyses for each protein and all ischemic stroke after adjustment for age, sex, and vascular risk factors are displayed in Figure 1 (for univariable results, please see Table S4). For acute-phase levels, 48 proteins were significantly and independently associated with ischemic stroke after correction for multiple testing (false discovery rate, *q* <0.05). At 3-month follow-up, 51 proteins were associated and at 7-year follow-up 50 proteins were independently associated with ischemic stroke. Most of the proteins were significantly elevated in cases compared to controls (marked in blue; acute: 42; 3 months, 49; 7 years, 48), and 34 (52%) were significantly elevated at all 3 time points. Few proteins were significantly lower in cases compared with controls (marked in red; acute phase: 6; 3 months: 2; 7 years: 2).

**Figure 2.**
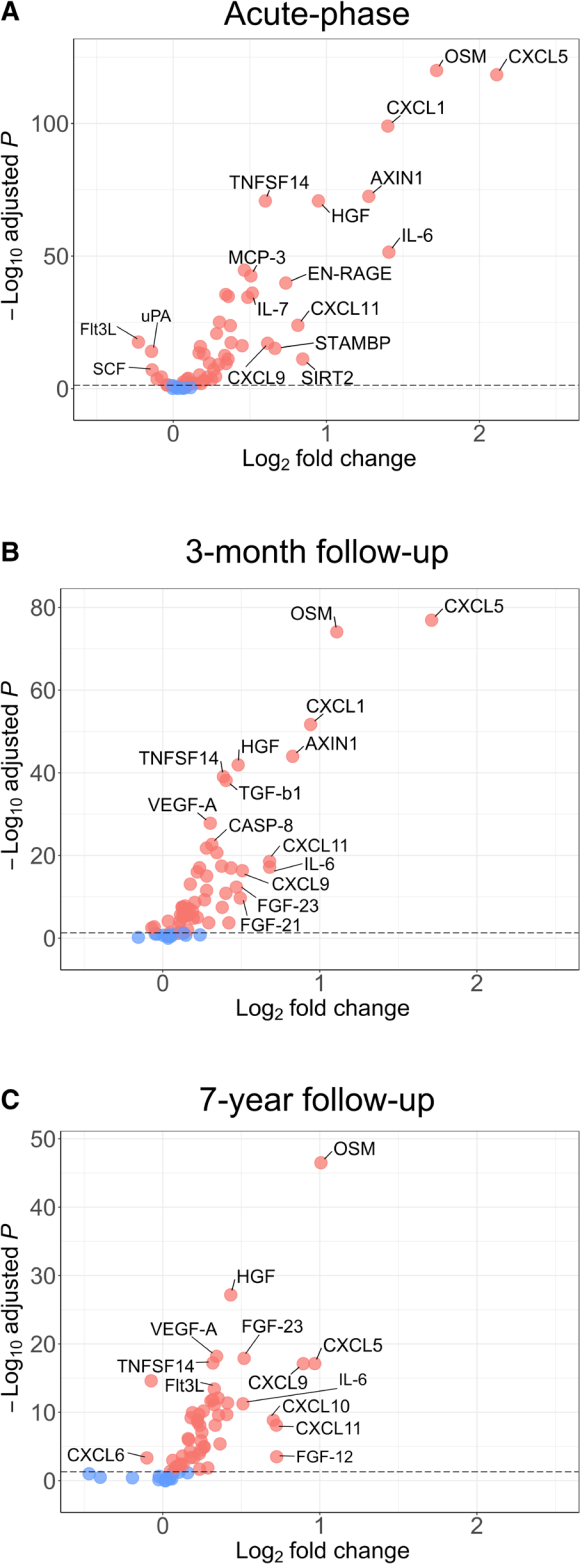
**Volcano plots showing differentially expressed proteins based on Normalized Protein eXpression values between ischemic stroke cases and controls. A**, Acute-phase protein levels; (**B**) 3-mo follow-up protein levels; and (**C**) 7-y follow-up protein levels. Each point represents one protein. A complete list of all protein names with corresponding UniProt identities are provided in Table S2. CXCL indicates CXC chemokine ligand; FGF, fibroblast growth factor; Flt3L, FMS-like tyrosine kinase 3 ligand; HGF, hepatocyte growth factor; IL, interleukin; OSM, oncostatin M; SCF, stem cell factor; and SIRT, sirtuin.

The proteins that had both the highest fold increase in cases compared to controls (ie, the most upregulated), and the lowest *P*-value in regression models were acute phase: CXCL5 (CXC chemokine ligand 5), OSM, CXCL1, AXIN1 (axis inhibition protein 1), IL-6, and HGF (hepatocyte growth factor; Figure 2A); 3-month: CXCL5, OSM, CXCL1, AXIN1, and HGF (Figure 2B); 7-year follow-up: CXCL5, OSM, CXCL9, HGF, and FGF (fibroblast growth factor)-23 (Figure 2C).

### Findings Were Internally Consistent and Robust in a Variety of Sensitivity Analyses

To examine reproducibility, we used a 2-step random-split approach for the acute and 3-month follow-up. In random sample 1, 39 proteins were significantly elevated, and 4 proteins were lower in cases compared to controls in the acute phase, and 43 proteins were significantly elevated and 1 was lower in cases at the 3-month follow-up (Table S5). The majority of associations were similar in random sample 2 (acute phase n=33 elevated and n=2 lowered; 3-month n=41 elevated and n=1 lowered). Those that did not make the significance threshold in one of the samples were directionally the same in both random sample sets.

We also conducted sensitivity analyses to exclude either cases with clinical signs of infection at the time of blood drawn (acute phase) or cases that experienced recurrent stroke or other vascular event during follow-up. The results were essentially unchanged in all of these analyses (Table S6).

It is of note that none of the proteins that were most elevated at the 7-year follow-up exhibited strong associations with age (Table S3). Nonetheless, to rule out potential confounding due to age, we repeated the 7-year regression analyses using a subset of controls matched for sex and age of cases at the 7-year follow-up (median age 63 years [interquartile range, 56–68]). The results were unchanged (eg, odds ratio [95% CI], CXCL5, 1.6 [1.4–1.8]; OSM, 5.4 [3.8–7.7]; CXCL9, 2.9 [2.1–4.1]; HGF, 9.2 [5.1–16.6]; FGF-23, 5.2 [3.1–8.7]; *q* <0.001 for all).

Given that the subgroup that participated in the 7-year follow-up had a different stroke subtype case-mix than the whole sample (Table), we analyzed protein levels from the acute phase and 3-month follow-up using just this subgroup of 223 cases. The results were largely unchanged, and the proteins that did not meet the significance thresholds were directionally the same as in the original analysis (Table S7).

**Table.**
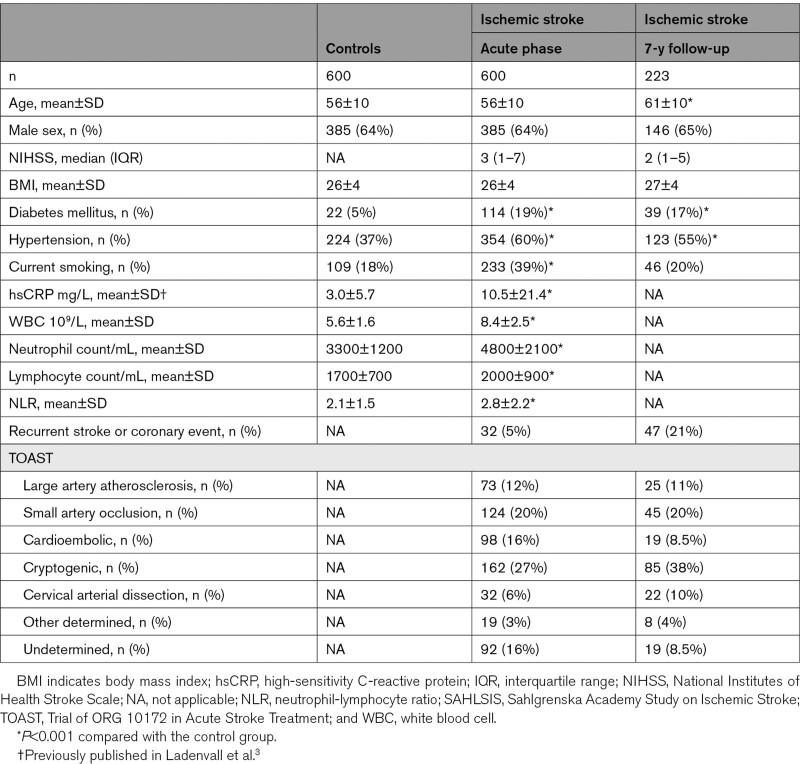
Characteristics of Ischemic Stroke Cases and Controls in SAHLSIS at Baseline and for a Subgroup at a 7-Year Follow-Up

### Subtype-Specific Associations

**Figure 3.**
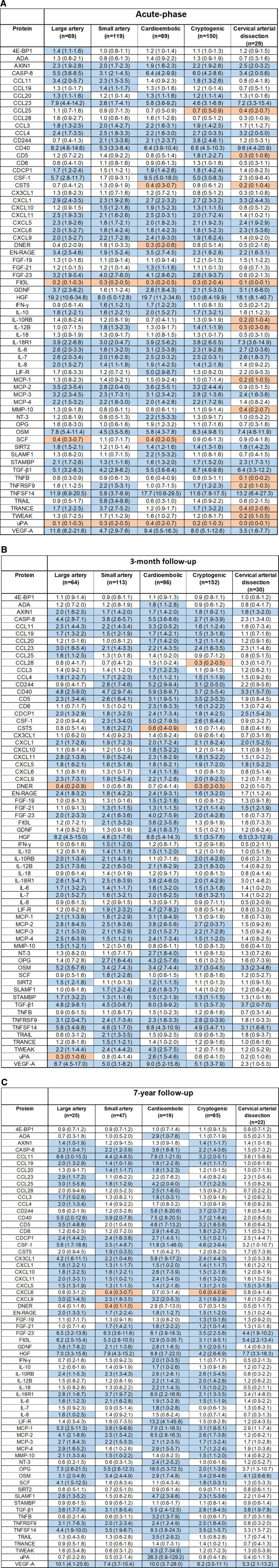
**Odds ratios and 95% CIs for ischemic stroke subtypes per one Normalized Protein eXpression value (ie, doubling of protein concentration) as compared to controls. A**, Acute-phase; (**B**) 3-mo follow-up; and (**C**) 7-y follow-up protein levels. Binary regressions were adjusted for age, sex, body mass index, diabetes, hypertension, smoking, and multiple testing (false discovery rate). White, nonsignificant (*q*>0.05); blue, significant (*q* <0.05) and elevated in cases; red, significant (*q* <0.05) and lower in cases compared with controls. A complete list of all protein names with corresponding UniProt identities are provided in Table S2.

Results from the multivariable binary logistic regression analyses (ie, case versus control) for each of the 4 main subtypes (LAA, SAO, cardioembolic and cryptogenic stroke) of ischemic stroke and the small group of cases with CeAD at each time point are presented in Figure 3. Subtype-specific volcano plots showing the fold difference in cases compared with controls at each time point are available in Figure S3. Overall, the pattern of the associations was similar across all subtypes in the acute phase. In total, 21 proteins were significantly and independently associated with stroke across all 5 subtypes: 19 were elevated (marked in blue) and 2 were lowered in cases compared with controls (in red; Figure 3). Several other proteins had the same direction of association in all subtypes; however, the significance threshold was not met in ≥1 subtypes. There were also several proteins with subtype-specific associations. CeAD had the largest number of downregulated proteins in the acute phase (in red; Figure 3A), and several of these proteins were significantly elevated in another subtype (eg, CD5 in cryptogenic stroke; IL-12B and TNFRSF9 in SAO and cryptogenic stroke; and TRANCE in LAA, SAO, and cryptogenic stroke).

At 3-month and 7-year follow-up, the majority of proteins remained significantly elevated in the 4 subtypes LAA, SAO, cardioembolic, and cryptogenic stroke. However, very few associations were observed in CeAD during follow-up (Figure 3 and Figure S3). In general, protein levels were most elevated in LAA and cardioembolic stroke across all time points. Exceptions of proteins most elevated in SAO stroke in the acute phase include CCL19, TRAIL, and TRANCE.

## Discussion

In this longitudinal explorative study of circulating inflammation-related proteins, we found over 30 proteins that were elevated in ischemic stroke cases compared with controls not only during the acute phase but also in the convalescent phase and at long-term follow-up. Although some proteins were elevated in all subtypes in the acute phase, and likely reflect a general response to cerebral ischemia, subtype-specific associations were also observed which could reflect underlying pathophysiological processes.

The majority of the proteins elevated in the acute phase after stroke remained elevated (though somewhat attenuated) during short- and long-term follow-up with respect to controls. Given that the 3-month and 7-year protein signatures should not be influenced by the ischemic event itself, we speculate that these proteins were elevated before cerebral injury and are candidate biomarkers of underlying inflammation. Examples include 3 of the most upregulated proteins in cases as compared to controls across time points: CXCL5, HGF, and OSM. These pleiotropic proteins have previously been implicated in atherosclerosis (OSM and CXCL5),^17,18^ endothelial barrier failure (OSM),^19^ and response to endothelial injury (HGF).^20^ In terms of stroke, one small study found CXCL5 to be elevated in the cerebral spinal fluid but not in serum within 24 hours after ischemic stroke,^21^ and elevated baseline levels of HGF were associated with an increased risk of incident stroke and coronary heart disease in a prospective cohort.^20^ As far as we are aware, plasma protein levels of OSM have not been analyzed in clinical ischemic stroke cohorts to date. Taken together, these proteins likely reflect underlying inflammation (eg, atherosclerosis), although they likely also partially reflect the response to the ischemic event per se as they were most elevated during the acute phase. For this group of proteins, studies with a prospective design could reveal novel prognostic markers for ischemic stroke.

In contrast to the set of proteins that remained elevated at follow-up, there was also a group that returned to control (or near control) levels. These proteins most likely primarily reflect the acute phase response. It is interesting to note that this group included SIRT2 (sirtuin 2), which has demonstrated roles in the response to hypoxia and ischemia-reperfusion injury^22^ and thus has more plausible relevance during the acute phase of ischemic stroke as compared to follow-up. Our data replicate and confirms data from a recent study where serum concentrations of SIRT2 were found to be elevated in 164 acute ischemic stroke cases relative to controls.^23^ It is of note that in our study, SIRT2 was one of the most upregulated proteins in CeAD in the acute phase which might reflect that hypoxia-induced inflammation, rather than other underlying inflammation, is a key component of the inflammatory response in this group. In line with this assumption, very few proteins remained elevated at follow-up in CeAD stroke.

Similar to the group of proteins discussed above, a small group of proteins were lower in the acute phase but returned to control levels during the follow-up and may thus also mainly reflect the acute-phase response. This group includes Flt3L, which was downregulated in all subtypes. Flt3L is important for dendritic cell differentiation and low Flt3L concentrations have been reported in patients with coronary artery disease^24^ compared to controls. One small study reported lower serum Flt3L concentrations in severe (NIHSS≥5; n=94) versus minor (NIHSS<5, n=53) ischemic stroke.^25^ In line with this, we found that acute Flt3L levels were inversely correlated with NIHSS (Pearson *r*=−0.25). Further studies are needed to evaluate whether some or a combination of these potential novel acute phase markers of ischemic stroke have predictive utility in terms of outcome after stroke.

Although protein levels were generally highest in LAA and cardioembolic stroke across time points, we observed interesting exceptions which could reflect subtype-specific processes. For example, TRAIL (or TNFSF10 [TNF-superfamily 10]) was significantly elevated only in the SAO subtype in the acute phase and at 3-month follow-up. This is in line with a study on 293 patients with acute ischemic stroke that found highest serum TRAIL levels in SAO stroke and lowest in cardioembolic stroke.^26^ Data from animal studies indicate that TRAIL may contribute to the pathophysiology of atherosclerosis and ischemic stroke.^27^ Another TNF superfamily member, TRANCE (or TNFSF11), was most elevated in SAO stroke in the acute phase. Circulating TRANCE has been linked to vascular calcification and arterial damage^28^ and elevated serum levels of TRANCE have been associated with incident cardiovascular disease (defined as ischemic stroke, transient ischemic attack, myocardial infarction, or vascular death).^29^

Another interesting subtype-specific association was SCF (stem cell factor). SCF plays a role in vascular repair and lower circulating levels of SCF have been demonstrated to associate with an increased risk of stroke and cardiovascular mortality^30^ and incident coronary events.^31^ Low SCF has also been associated with more severe carotid disease, less fibrous atherosclerotic plaques, and increased incidence of heart failure.^31^ In line with this, we observed lowered SCF levels in LAA and cardioembolic stroke, but not in SAO, cryptogenic stroke, or CeAD.

Strengths in the current study are inclusion of consecutive and well-characterized, young (<70 years) ischemic stroke cases, in whom there are less potentially confounding comorbidities than in older onset ischemic stroke cases. Furthermore, standardized blood sampling was repeated in the same individuals across fixed time points over the course of a long-term follow-up. The protein levels from the different time points and from the controls were measured simultaneously, using a technology with high specificity. We also performed a variety of sensitivity analyses where we found the results to be internally consistent and robust. Our study also has important limitations. First, is the lack of an independent replication cohort. However, in lieu of this, we performed a 2-step random-split approach where we found the results to be internally reproducible. It is also of note that many of these proteins have recently emerged as novel biomarkers of cardiovascular disease and many have existing experimental data supporting a role in ischemic stroke. Second, the case-control design of our study prevents determination as to whether the observed changes in protein levels are causal of, or secondary to, the ischemic stroke event. In the future, prospective studies are required to elucidate whether some of these proteins were elevated before stroke. Third, the stroke survivors who attended the 7-year follow-up visit had milder strokes than the remainder of the stroke cohort, which may limit the generalizability of our 7-year results. We are also aware that 7 years is a very long time after stroke and we cannot exclude the influence of unaccounted confounding (eg, progressive small vessel disease, comorbidities, or medications). It is of note that most proteins had negligible correlation with age and an analysis using only a subset of controls matched for sex and age of cases at 7-year follow-up yielded similar results. Fourth, the clinical workup on underlying mechanisms for ischemic stroke has improved since our data was gathered in 2003. However, the proportion of cases with incomplete evaluation in this study was modest (11%).

In conclusion, we report a large number of inflammation-related proteins that are elevated in ischemic stroke over a long-term follow-up study and also provide data for the 4 main etiological subtypes as well as cervical arterial dissections. The majority of these proteins have not been studied in clinical ischemic stroke cohorts, although many have been implicated in cardiovascular diseases. Validation in independent ischemic stroke cohorts and further studies evaluating the potential importance of these proteins for risk or prognostication are warranted.

## Article Information

### Acknowledgments

We thank research nurse Ingrid Eriksson for her excellent assistance in recruiting the study participants and for conducting the follow-up study. Furthermore, we are grateful to our study participants without whom this work would not have been possible.

### Sources of Funding

The Swedish Heart and Lung Foundation (20190203); the Swedish Research Council (2021-01114). The study was financed by grants from the Swedish state under the agreement between the Swedish government and the country councils, the ALF-agreement (ange anslagsnummer) (ALFGBG-720081). the King Gustaf V:s and Queen Victoria´s Freemason's Foundation; the Rune and Ulla Amlövs Foundation; the John and Brit Wennerström Foundation; the Per-Olof Ahl Foundation; and the Gothenburg Foundation for Neurological Research.

### Disclosures

Dr Jern has full access to all the data in the study and takes responsibility for its integrity and the data analysis. The other authors report no conflicts.

### Supplemental Material

STROBE Checklist

Supplemental Methods

Figures S1–S2

Tables S1–S7

## Supplementary Material


