# Supplementary material for: Longitudinal Study Reveals Long-Term Proinflammatory Proteomic Signature After Ischemic Stroke Across Subtypes
Source: Stroke. 2022 Jun 10;53(9):2847–58. doi: 10.1161/STROKEAHA.121.038349 (PMC9389938; doi:10.1161/STROKEAHA.121.038349)
Supplement: Supplementary file 1 [file str-53-2847-s001.pdf]

SUPPLEMENTAL MATERIAL

Stroke

Longitudinal study reveals long-term proinflammatory proteomic signature after ischemic stroke across subtypes

Tara M. Stanne[1], Annelie Angerfors[1], Björn Andersson[2], Cecilia Brännmark[1], Lukas Holmegaard[3,4], Christina Jern[1, 5]

[1] Institute of Biomedicine, Department of Laboratory Medicine, the Sahlgrenska Academy, University of Gothenburg, Gothenburg, Sweden
[2] Bioinformatics Core Facility, University of Gothenburg, Gothenburg, Sweden
[3] Institute of Neuroscience and Physiology, Department of Clinical Neuroscience, the Sahlgrenska Academy, University of Gothenburg, Gothenburg, Sweden
[4] Region Västra Götaland, Sahlgrenska University Hospital, Department of Neurology, Gothenburg, Sweden
[5] Region Västra Götaland, Sahlgrenska University Hospital, Department of Clinical Genetics and Genomics, Gothenburg, Sweden

Corresponding author: Tara Stanne. E-mail: tara.stanne@gu.se

STROBE Statement—checklist of items that should be included in reports of cohort studies

	Item No	Recommendation	Reported on page
**Title and abstract**	1	(*a*) Indicate the study's design with a commonly used term in the title or the abstract	1
		(*b*) Provide in the abstract an informative and balanced summary of what was done and what was found	1
**Introduction**			
Background/rationale	2	Explain the scientific background and rationale for the investigation being reported	3
Objectives	3	State specific objectives, including any prespecified hypotheses	4
**Methods**			
Study design	4	Present key elements of study design early in the paper	4
Setting	5	Describe the setting, locations, and relevant dates, including periods of recruitment, exposure, follow-up, and data collection	4
Participants	6	(*a*) *Cohort study*—Give the eligibility criteria, and the sources and methods of selection of participants. Describe methods of follow-up<br>*Case-control study*—Give the eligibility criteria, and the sources and methods of case ascertainment and control selection. Give the rationale for the choice of cases and controls<br>*Cross-sectional study*—Give the eligibility criteria, and the sources and methods of selection of participants	4 and 5
		(*b*) *Cohort study*—For matched studies, give matching criteria and number of exposed and unexposed<br>*Case-control study*—For matched studies, give matching criteria and the number of controls per case	4 and 5
Variables	7	Clearly define all outcomes, exposures, predictors, potential confounders, and effect modifiers. Give diagnostic criteria, if applicable	5
Data sources/ measurement	8*	For each variable of interest, give sources of data and details of methods of assessment (measurement). Describe comparability of assessment methods if there is more than one group	6
Bias	9	Describe any efforts to address potential sources of bias	7
Study size	10	Explain how the study size was arrived at	na
Quantitative variables	11	Explain how quantitative variables were handled in the analyses. If applicable, describe which groupings were chosen and why	7
Statistical methods	12	(*a*) Describe all statistical methods, including those used to control for confounding	7
		(*b*) Describe any methods used to examine subgroups and interactions	7
		(*c*) Explain how missing data were addressed	na
		(*d*) *Cohort study*—If applicable, explain how loss to follow-up was addressed<br>*Case-control study*—If applicable, explain how matching of cases and controls was addressed<br>*Cross-sectional study*—If applicable, describe analytical methods	na

		taking account of sampling strategy	
		(*e*) Describe any sensitivity analyses	8
**Results**			
Participants	13*	(a) Report numbers of individuals at each stage of study—eg numbers potentially eligible, examined for eligibility, confirmed eligible, included in the study, completing follow-up, and analysed	7, Table 1 Online Table S1
		(b) Give reasons for non-participation at each stage	na
		(c) Consider use of a flow diagram	na
Descriptive data	14*	(a) Give characteristics of study participants (eg demographic, clinical, social) and information on exposures and potential confounders	Table 1
		(b) Indicate number of participants with missing data for each variable of interest	7, Online Table S1 and S2
		(c) *Cohort study*—Summarise follow-up time (eg, average and total amount)	5
Outcome data	15*	*Cohort study*—Report numbers of outcome events or summary measures over time	na
		Case-control study—Report numbers in each exposure category, or summary measures of exposure	7
		Cross-sectional study—Report numbers of outcome events or summary measures	na
Main results	16	(*a*) Give unadjusted estimates and, if applicable, confounder-adjusted estimates and their precision (eg, 95% confidence interval). Make clear which confounders were adjusted for and why they were included	8, Figure 1 and 3; Online S4
		(*b*) Report category boundaries when continuous variables were categorized	na
		(*c*) If relevant, consider translating estimates of relative risk into absolute risk for a meaningful time period	na
Other analyses	17	Report other analyses done—eg analyses of subgroups and interactions, and sensitivity analyses	10-11
**Discussion**			
Key results	18	Summarise key results with reference to study objectives	12
Limitations	19	Discuss limitations of the study, taking into account sources of potential bias or imprecision. Discuss both direction and magnitude of any potential bias	15
Interpretation	20	Give a cautious overall interpretation of results considering objectives, limitations, multiplicity of analyses, results from similar studies, and other relevant evidence	13-15
Generalisability	21	Discuss the generalisability (external validity) of the study results	16
**Other information**			
Funding	22	Give the source of funding and the role of the funders for the present study and, if applicable, for the original study on which the present article is based	17

*Give information separately for cases and controls in case-control studies

Note: An Explanation and Elaboration article discusses each checklist item and gives methodological background and published examples of transparent reporting. The STROBE checklist is best used in conjunction with this article (freely available on the Web sites of PLoS Medicine at http://www.plosmedicine.org/, Annals of Internal Medicine at http://www.annals.org/, and Epidemiology at http://www.epidem.com/). Information on the STROBE Initiative is available at www.strobe-statement.org.

SUPPLEMENTAL METHODS

Study population

Index stroke severity was scored as maximum severity within the first 7 days after hospital admission using the Scandinvian Stroke Scale (SSS). The SSS is similar to the National Institutes of Health Stroke Scale (NIHSS), but was more commonly used in Sweden during the first years of recruitment to the *Sahlgrenska Academy Study on Ischemic Stroke* (*SAHLSIS*). To facilitate comparisons with other studies, we have converted all individual SSS scores to NIHSS scores using an algorithm (Gray et al, 2009).[12] Stroke subtypes were defined according to the Trial of Org 10172 in Acute Stroke Treatment (TOAST) criteria (Adams et al, 1993).[13] It is of note that, as this study included stroke cases aged 18-69 years, the proportion of cases that had imaging of vessels other than carotid doppler ultrasound was relatively high compared to standard clinical routine at the time of inclusion, and 35% additionally underwent magnetic resonance cerebral angiography, 19% transcranial doppler ultrasound, and 11% conventional cerebral angiography.

Definition of vascular risk factors

Information regarding vascular risk factors was registered at inclusion for controls and at the 3-month and 7-year follow-up for cases by examinations and a structured questionnaire, as described (Jood et al, 2005).[11] In brief, hypertension was defined as pharmacological treatment for hypertension and/or systolic blood pressure ≥160 mm Hg, and/or diastolic blood pressure ≥90 mm Hg. Diabetes mellitus was defined as dietary or pharmacological treatment and/or fasting plasma glucose ≥7.0 mmol/L or fasting blood glucose ≥6.1 mmol/L. Smoking habit was coded as current versus never or former (smoking cessation at least one year before inclusion in the study).

Blood sampling and protein measurement

All blood samples from cases in the acute phase were drawn by nurses at four stroke units; i.e. two stroke units at the Sahlgrenska University Hospital, one unit at the Södra Älvsborg's Hospital and one unit at Skaraborg's Hospital. All patients attended 3-month follow up visits at outpatient clinics at the same hospital they attended at inclusion between 1998 and 2003. A subgroup of 223 cases recruited at the main stroke unit at the Sahlgrenska University Hospital attended a 7-year follow up visit at the same outpatient clinic. Controls were geographically matched to cases and had their study visits at the same outpatient clinic as the respective case had the 3-month follow-up visit between 1999 and 2004. All blood samples were collected and handled using the same protocol throughout the study period.

In the present study, plasma levels of inflammation-related proteins were analyzed using a multiplexed assay, the Proseek Multiplex Inflammation I assay (Olink Bioscience, Uppsala, Sweden), which uses proximity extension assay (PEA) technology and is described in detail elsewhere (Assarsson et al, 2014).[16] In brief, each protein is specifically targeted by one pair of antibodies labeled with complementary oligonucleotides. Upon specific antibody-antigen binding, the complementary oligonucleotides hybridize due to their close proximity. Standard real-time polymerase chain reaction (PCR) is then used to generate and quantify the resulting protein specific target sequences. PCR readouts are normalized by an extension control added to each sample and an inter-plate control on each plate. The final PEA output is given as Normalized Protein eXpression (NPX) values, an arbitrary unit given on a log2 scale where a higher value corresponds to a higher protein level. Of note, the NPX level corresponds to relative levels, i.e. absolute protein concentrations are not determined.

Statistical analyses

Of the 92 proteins, 65 showed a pre-specified call rate of >80% and were included in further analyses. Of the included proteins, 49 did not have any samples below the limit of detection (LOD) in controls or cases at any of the 3 time points; 5 proteins had 1 sample below the LOD (FGF-23, IL-6, IL-10, MCP-4, and SIRT2), 2 proteins had <10 samples below the LOD (CCL28 and OSM); 3 had 10-50 samples below the LOD (CASP-8, IL-7, and TNFSF14) and 6 had >50 samples below the LOD (AXIN1, EN-RAGE, GDNF, IFN-γ, MCP-3, and SLAMF1), for details see Table S1 in the Online Supplement.

SUPPLEMENTAL FIGURES

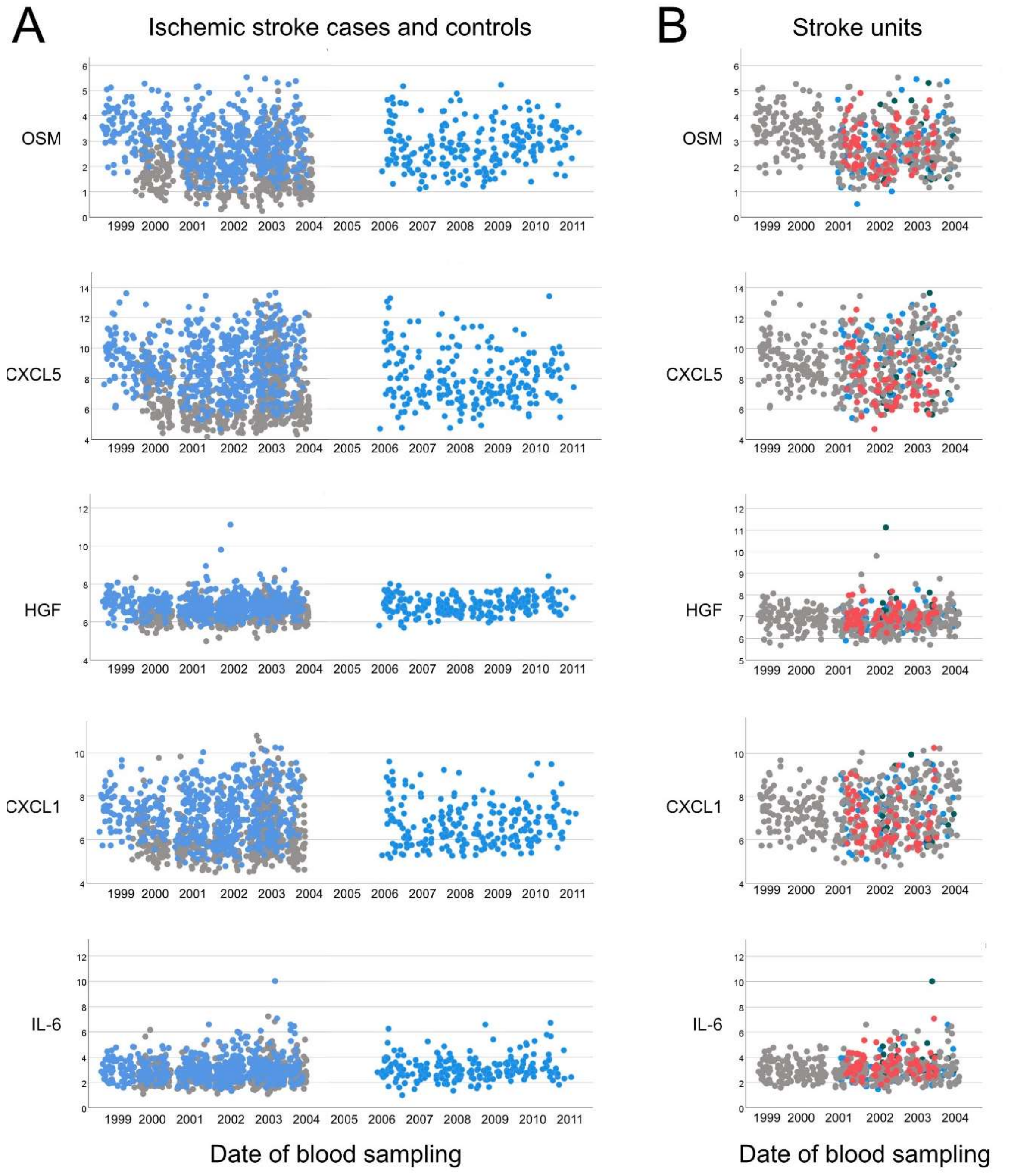

Figure S1. Scatter plots for five representative proteins showing protein levels (NPX values) versus date of blood sampling. In total 600 ischemic stroke cases and 600 age, sex and geographically matched controls were included in SAHLSIS at four stroke units between 1998 and 2003 and a subset of 223 patients included at the main site (Sahlgrenska University Hospital) attended a 7-year follow-up between 2005-2011. Controls attended the same outpatient clinic as their respective cases for blood draw between 1999 and 2004. Standardized blood sampling was performed on all occasions. Plasma was aliquoted and stored at -80C pending analysis. A) 3-month and 7-year levels of ischemic stroke cases (blue) and controls (grey); B) 3-month levels of ischemic stroke cases colour overlayed for the four sites of blood sampling: Sahgrenska University Hospital, grey and dark green; Södra Älvsborg's Hospital, red; and Skaraborg's Hospital, blue.

A

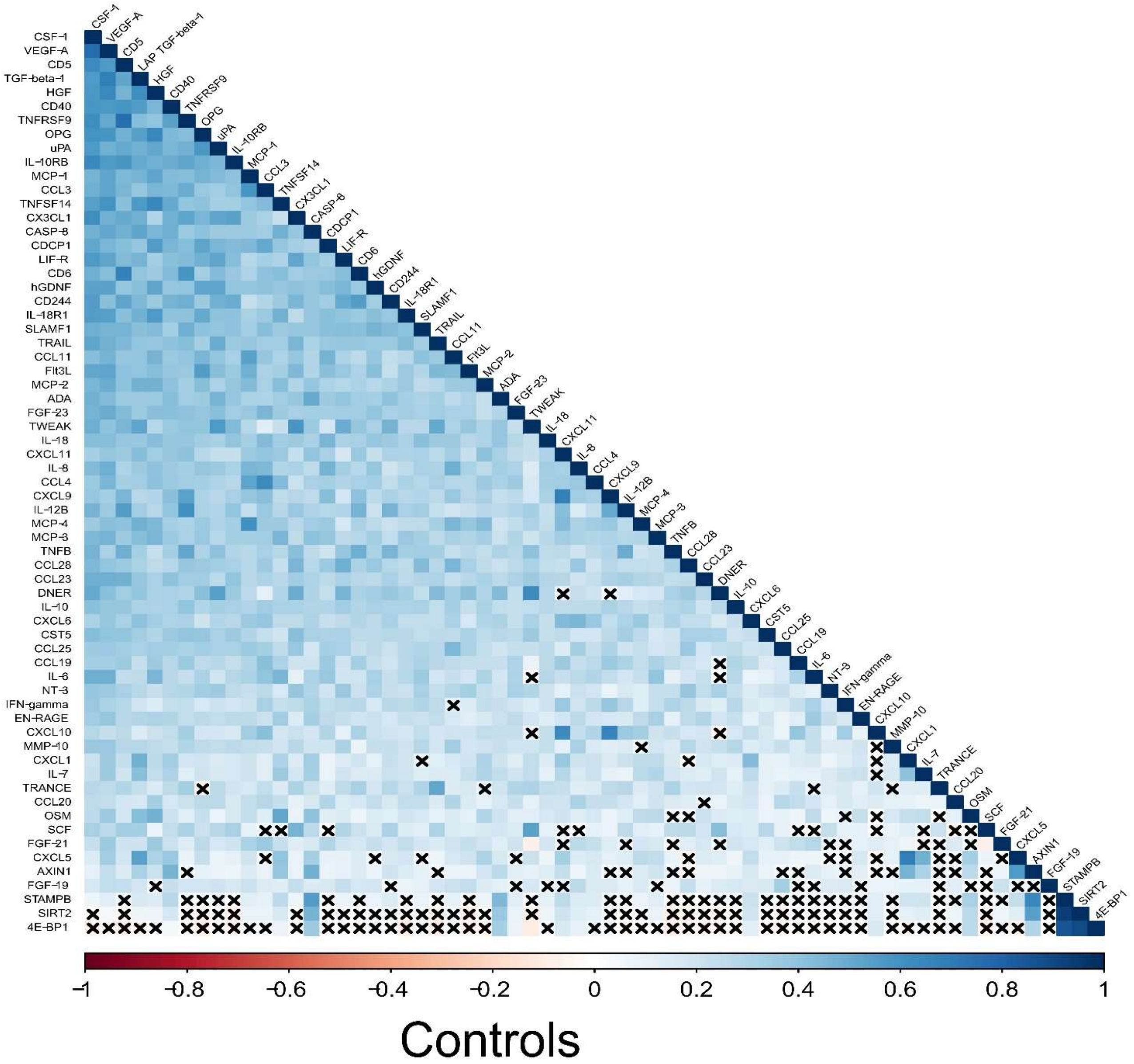

Figure S2. Pearson's correlation coefficients (r) between proteins in **A**) controls. Correlations with $p<0.05$ are marked in colour and those with $p>0.05$ are indicated with an x. Positive correlations ($r>0$) between protein levels are marked blue and inverse correlations ($r<0$) are marked red. The strongest correlations were between three proteins: 4E-BP1, SIRT2 and STAMP ($r>0.85$).

B

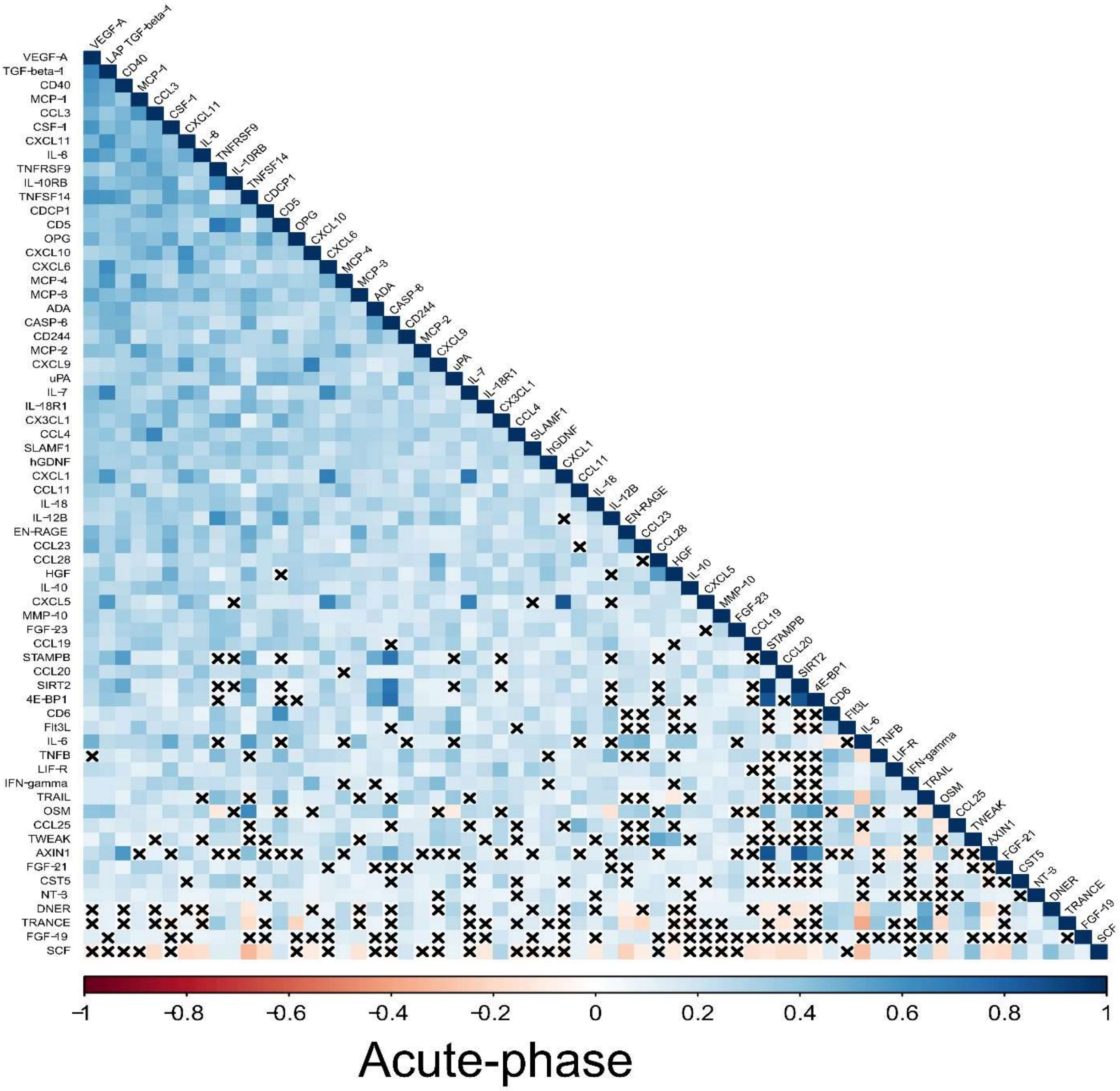

Figure S2. Pearson's correlation coefficients (r) between proteins in **B**) ischemic stroke cases in the acute-phase. Correlations with $p<0.05$ are marked in colour and those with $p>0.05$ are indicated with an x. Positive correlations ($r>0$) between protein levels are marked blue and inverse correlations ($r<0$) are marked red. The strongest correlations were between three proteins: 4E-BP1, SIRT2 and STAMP ($r>0.85$).

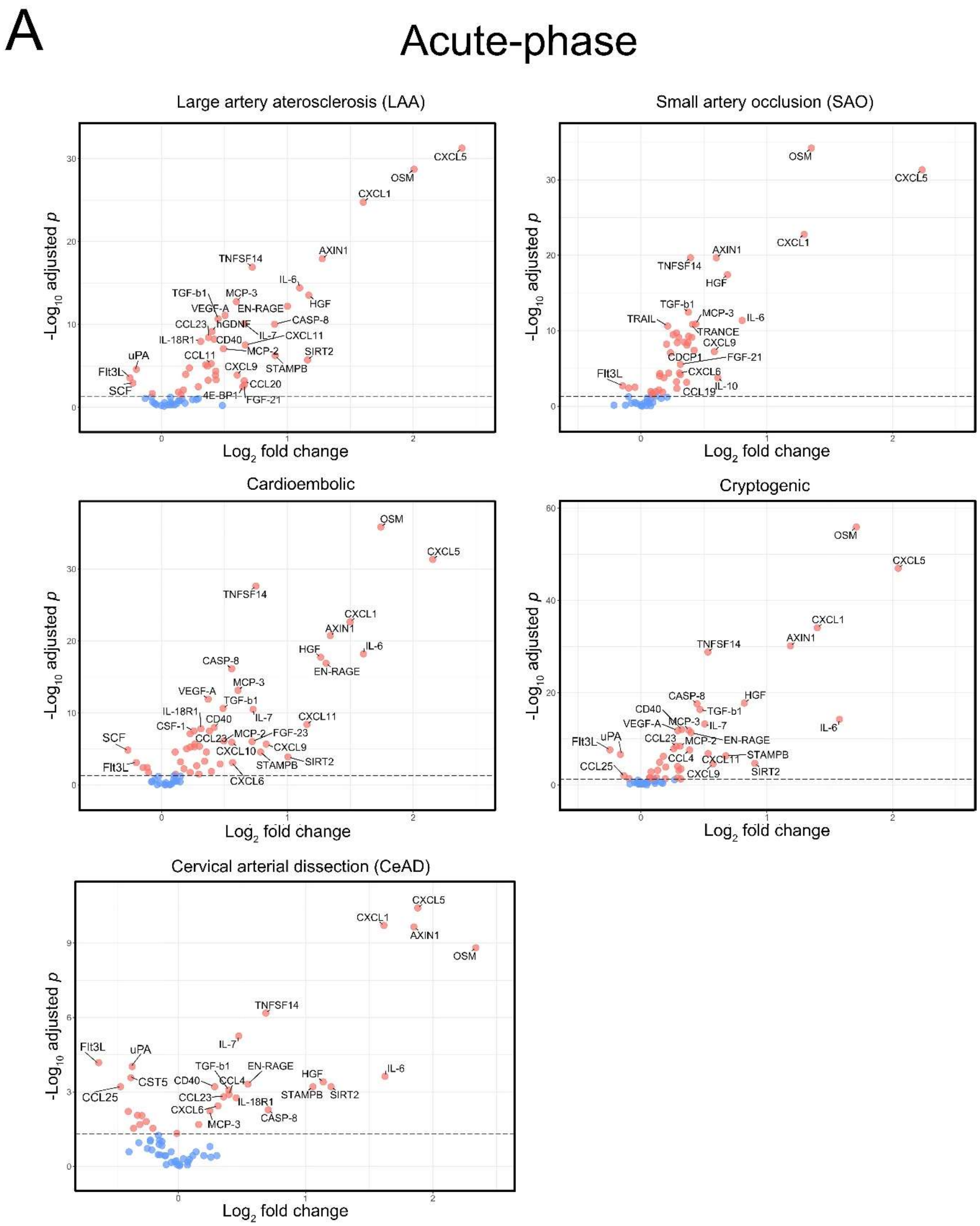

Figure S3. Volcano plots showing differentially-expressed proteins based on normalized protein expression (NPX) values between ischemic stroke cases and controls for each of the four main subtypes and the small group of cervical arterial dissections. **A**) Acute-phase. Each point represents one protein. The FDR corrected significance threshold is indicated. Red circles, significant (adjusted $p < 0.05$); blue circles, not significant.

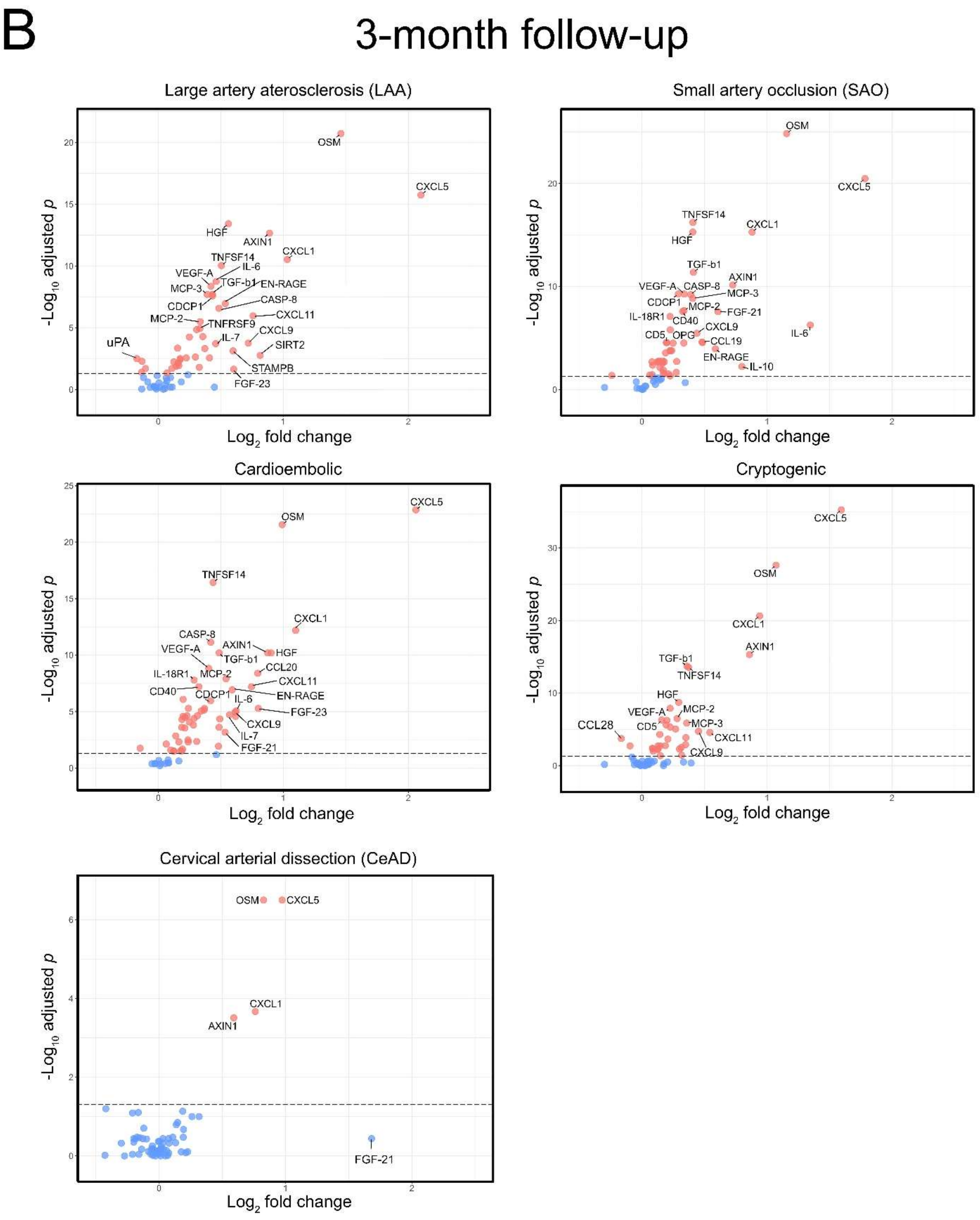

Figure S3. Volcano plots showing differentially-expressed proteins based on normalized protein expression (NPX) values between ischemic stroke cases and controls for each of the four main subtypes and the small group of cervical arterial dissections. **B**) 3-month follow-up. Each point represents one protein. The FDR corrected significance threshold is indicated. Red circles, significant (adjusted *p* <0.05); blue circles, not significant.

C 7-year follow-up

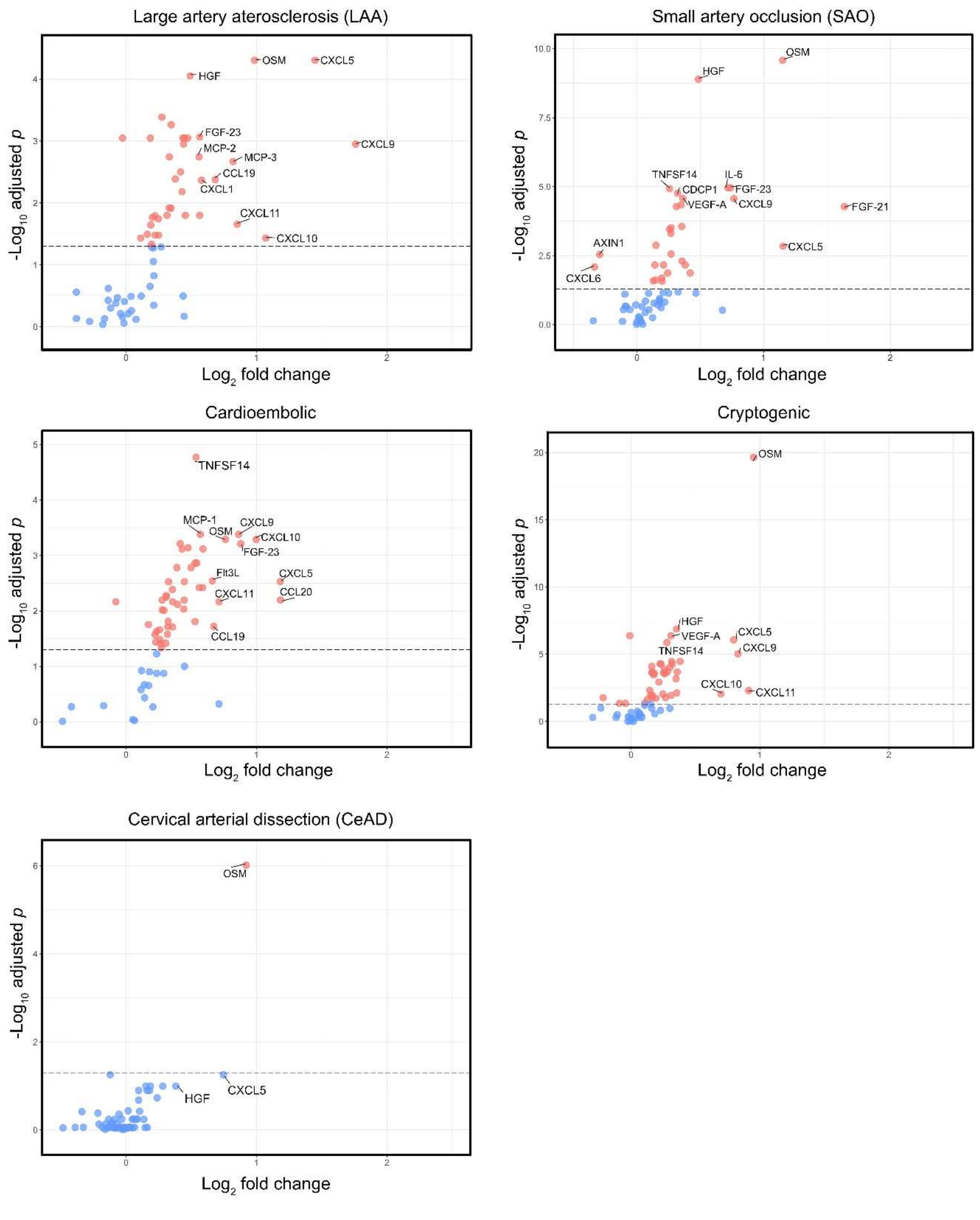

Figure S3. Volcano plots showing differentially-expressed proteins based on normalized protein expression (NPX) values between ischemic stroke cases and controls for each of the four main subtypes and the small group of cervical arterial dissections. **C**) 7-year follow-up. Each point represents one protein. The FDR corrected significance threshold is indicated. Red circles, significant (adjusted $p<0.05$); blue circles, not significant.

Table S1: Year and site of blood sampling in the *Sahlgrenska Academy Study on Ischemic Stroke* (*SAHLSIS*).

		**Ischemic stroke cases**					**Population controls**			
	**Admission Year**	**All**	**Sahlgrenska University Hospital** [1]		**Södra Älvsborg's Hospital**	**Skaraborg's Hospital**	**All**	**Sahlgrenska University Hospital**	**Södra Älvsborg's Hospital**	**Skaraborg's Hospital**
**Included**	**1998**	34	34	-	-	-	0	-	-	-
	**1999**	65	65	-	-	-	60	60	-	-
	**2000**	80	66	-	11	3	100	100	-	-
	**2001**	153	82	5	26	40	89	89	-	-
	**2002**	157	91	9	28	29	118	79	21	18
	**2003**	111	73	10	16	12	217	92	60	65
	**2004**	-	-	-	-	-	16	15	-	1
	**Total**	600	411	24	81	84	600	435	81	84
**Missing plasma acute / control**	**1998**	3	3	-	-	-	0	-	-	-
	**1999**	1	1	-	-	-	2	2	-	-
	**2000**	0	0	-	0	0	4	4	-	-
	**2001**	7	5	0	0	2	5	5	-	-
	**2002**	16	12	2	1	1	11	7	1	3
	**2003**	12	7	5	0	0	20	18	1	1
	**2004**	-	-	-	-	-	2	2	-	0
	**Total**	39	28	7	1	3	44	38	2	4
**Missing plasma 3-month**	**1998**	5	5	-	-	-	NA	NA	NA	NA
	**1999**	4	4	-	-	-	NA	NA	NA	NA
	**2000**	9	8	-	0	1	NA	NA	NA	NA
	**2001**	12	5	1	3	3	NA	NA	NA	NA
	**2002**	13	10	1	1	1	NA	NA	NA	NA
	**2003**	8	6	1	0	1	NA	NA	NA	NA
	**2004**	-	-	-	-	-	NA	NA	NA	NA
	**Total**	51	38	3	4	6	NA	NA	NA	NA

In total 600 ischemic stroke cases and 600 age, sex and geographically matched controls were included. The number of cases and controls include per site, as well as the number of missing plasma samples included per site and time-point is provided.

[1]Blood samples were collected from patients during the acute-phase at two stroke units at the Sahlgrenska University Hospital. Follow-up visits for cases and baseline visits for controls were at a single outpatient clinic at the Sahlgrenska University Hospital. A subgroup of 223 patients from the main Sahlgrenska University Hospital site attended a 7-year follow-up at the same outpatient clinic and no samples are missing.

SUPPLEMENTAL TABLES

Table S2. Protein names and number of samples below the limit of detection (LOD).

Protein	Gene	Protein name	Nr samples below LOD
4E-BP1	4E-BP1	Eukaryotic translation initiation factor 4E-binding protein 1	0
ADA	ADA	Adenosine Deaminase	0
AXIN1	AXIN1	Axin-1	51
CASP-8	CASP8	Caspase-8	26
CCL11	CCL11	Eotaxin	0
CCL19	CCL19	C-C motif chemokine 19	0
CCL20	CCL20	C-C motif chemokine 20	0
CCL23	CCL23	C-C motif chemokine 23	0
CCL25	CCL25	C-C motif chemokine 25	0
CCL28	CCL28	C-C motif chemokine 28	4
CCL3	CCL3	C-C motif chemokine 3	0
CCL4	CCL4	C-C motif chemokine 4	0
CD244	CD244	Natural killer cell receptor 2B4	0
CD40	CD40	CD40L receptor	0
CD5	CD5	T-cell surface glycoprotein CD5	0
CD6	CD6	T cell surface glycoprotein CD6 isoform	0
CDCP1	CDCP1	CUB domain-containing protein 1	0
CSF-1	CSF1	Macrophage colony-stimulating factor 1	0
CST5	CST5	Cystatin D	0
CX3CL1	CX3CL1	Fractalkine	0
CXCL1	CXCL1	C-X-C motif chemokine 1	0
CXCL10	CXCL10	C-X-C motif chemokine 10	0
CXCL11	CXCL11	C-X-C motif chemokine 11	0
CXCL5	CXCL5	C-X-C motif chemokine 5	0
CXCL6	CXCL6	C-X-C motif chemokine 6	0
CXCL9	CXCL9	C-X-C motif chemokine 9	0
DNER	DNER	Delta and Notch-like epidermal growth factor-related receptor	0
EN-RAGE	S100A12	S100 Calcium Binding Protein A12	71
FGF-19	FGF-19	Fibroblast growth factor 19	0
FGF21	FGF21	Fibroblast growth factor 21	0
FGF-23	FGF23	Fibroblast growth factor 23	1
Flt3L	Flt3L	Fms-related tyrosine kinase 3 ligand	0
GDNF	GDNF	Glial cell line-derived neurotrophic factor	159
HGF	HGF	Hepatocyte growth factor	0
IFN-γ	IFNG	Interferon gamma	381

Table S2 Continued. Protein names and number of samples below the limit of detection (LOD).

Protein	Gene	Protein name	Nr samples below LOD
IL10	IL10	Interleukin-10	1
IL-10RB	IL10RB	Interleukin-10 receptor subunit beta	0
IL-12B	IL12B	Interleukin-12 subunit beta	0
IL-18	IL18	Interleukin-18	0
IL-18R1	IL18R1	Interleukin-18 receptor 1	0
IL6	IL6	Interleukin-6	1
IL-7	IL7	Interleukin-7	45
IL-8	CXCL8	Interleukin-8	0
LIF-R	LIFR	Leukemia inhibitory factor receptor	0
MCP-1	CCL2	Monocyte chemotactic protein 1	0
MCP-2	CCL8	Monocyte chemotactic protein 2	0
MCP-3	CCL7	Monocyte chemotactic protein 3 /C-C Motif Chemokine Ligand 7	377
MCP-4	CCL13	Monocyte chemotactic protein 4	1
MMP-10	MMP10	Matrix metalloproteinase-10	0
NT-3	NTF3	Neurotrophin-3	0
OPG	TNFRSF11B	Osteoprotegerin /TNF Receptor Superfamily Member 11b	0
OSM	OSM	Oncostatin-M	9
SCF	KITLG	Stem cell factor / KIT Ligand	0
SIRT2	SIRT2	SIR2-like protein 2	1
SLAMF1	SLAMF1	Signaling lymphocytic activation molecule	82
STAMBP	STAMBP	STAM-binding protein	0
TGF-β1	TGFB1	Latency-associated peptide transforming growth factor beta-1	0
TNFB	LTA	TNF-beta / Lymphotoxin-alpha	0
TNFRSF9	TNFRSF9	Tumor necrosis factor receptor superfamily member 9	0
TNFSF14	TNFSF14	Tumor necrosis factor ligand superfamily member 14	14
TRAIL	TNFSF10	TNF-related apoptosis-inducing ligand	0
TRANCE	TNFSF11	TNF-related activation-induced cytokine	0
TWEAK	TNFSF12	Tumor necrosis factor Ligand superfamily, member 12	0
uPA	PLAU	Urokinase-type plasminogen activator	0
VEGF-A	VEGFA	Vascular endothelial growth factor A	0

Table S3. Pearson's correlation coefficients (*r*) for each protein and clinical variables in controls and in ischemic stroke cases in the acute-phase.

	Controls					Ischemic Stroke, acute-phase						
	Age	hsCRP	WBC	NLR	BMI	Age	hsCRP	WBC	NLR	BMI	NIHSS	Days
Age	1	0.148*	0.043	-0.030	0.182*	1	0.086*	-0.004	0.046	0.137*	0.002	-0.069
hsCRP	0.148*	1	0.246*	0.218*	0.173*	0.086*	1	0.153*	0.403*	0.031	0.416*	-0.004
WBC	0.043	0.246*	1	0.203*	0.117*	-0.004	0.153*	1	0.165*	0.063	0.194*	-0.017
NLR	-0.030	0.218*	0.203*	1	0.071	0.046	0.403*	0.165*	1	-0.055	0.251*	-0.086
BMI	0.182*	0.173*	0.117*	0.071	1	0.137*	0.031	0.063	-0.055	1	-0.041	0.023
NIHSS	NA	NA	NA	NA	NA	0.002	0.416*	0.194*	0.251*	-0.041	1	-0.027
Days	-0.045	0.009	-0.042	-0.008	0.024	-0.069	-0.004	-0.017	-0.086	0.023	-0.027	1
4E-BP1	-0.034	-0.045	0.042	0.049	0.042	-0.055	0.063	-0.007	0.024	-0.009	0.057	-0.009
ADA	0.011	0.005	-0.018	0.113*	0.077	-0.076	0.157*	-0.015	0.004	0.035	0.016	-0.036
AXIN1	0.063	0.047	0.051	0.055	0.076	-0.056	0.190*	0.049	0.046	-0.012	0.072	0.000
CASP-8	0.154*	0.086*	0.108*	0.102*	0.075	-0.054	0.148*	0.039	0.077	-0.049	0.090*	-0.013
CCL11	0.360*	-0.027	0.056	-0.062	-0.070	0.233*	-0.136*	0.009	-0.255*	-0.021	-0.134*	0.038
CCL19	0.138*	0.092*	0.067	-0.010	0.225*	0.102*	0.171*	-0.055	-0.137*	0.141*	-0.009	0.031
CCL20	0.049	0.111*	0.113*	0.045	0.090*	0.018	0.299*	0.140*	0.068	0.021	0.128*	-0.003
CCL23	0.104*	0.174*	0.105*	0.004	-0.043	0.020	0.437*	0.219*	0.290*	-0.057	0.293*	-0.065
CCL25	0.232*	0.032	0.053	-0.002	0.000	0.137*	-0.149*	-0.014	-0.194*	-0.050	-0.174*	0.051
CCL28	0.146*	0.033	0.040	-0.067	-0.155*	0.097*	0.005	0.027	-0.079	-0.110*	0.070	0.045
CCL3	0.224*	0.100*	0.128*	0.010	0.161*	0.113*	0.225*	0.021	0.060	0.110*	0.065	0.061
CCL4	0.127*	0.014	0.067	0.013	0.140*	0.025	0.110*	0.034	0.059	0.054	0.076	0.022
CD244	0.032	-0.006	-0.028	0.016	0.089*	-0.087*	-0.054	-0.106*	-0.114*	0.061	-0.041	0.028
CD40	0.261*	0.115*	0.057	0.001	0.043	0.092*	0.286*	0.083	0.052	0.023	0.091*	-0.041
CD5	0.170*	0.061	0.149*	-0.074	0.032	-0.016	-0.071	-0.011	-0.269*	0.024	-0.116*	0.023
CD6	0.121*	0.049	0.057	-0.070	0.114*	-0.048	-0.208*	-0.030	-0.364*	0.095*	-0.138*	0.018
CDCP1	0.497*	0.179*	0.123*	0.003	0.166*	0.336*	0.207*	0.045	-0.012	0.075	0.096*	0.023
CSF-1	0.178*	0.276*	0.145*	0.074	0.074	0.057	0.408*	0.147*	0.214*	0.061	0.269*	-0.006
CST5	0.249*	0.092*	-0.049	-0.018	-0.056	0.160*	-0.040	-0.130*	-0.074	-0.088*	-0.114*	-0.047
CX3CL1	0.140*	-0.057	-0.142*	-0.006	-0.152*	0.087*	-0.023	-0.056	0.065	-0.088*	0.009	-0.023
CXCL1	0.122*	0.077	-0.031	0.057	0.046	-0.021	0.184*	0.128*	0.024	-0.061	0.070	-0.005
CXCL10	0.156*	0.082*	-0.035	0.051	0.083*	0.086*	0.170*	-0.103*	-0.019	0.070	0.013	0.097*
CXCL11	0.160*	0.106*	-0.018	0.122*	0.085*	0.012	0.238*	0.039	0.034	-0.026	0.034	0.082
CXCL5	0.194*	0.084*	0.049	0.024	0.048	0.027	0.062	0.127*	-0.070	-0.012	0.023	-0.007
CXCL6	0.113*	-0.037	-0.092*	0.008	0.061	-0.021	0.079	0.006	-0.032	0.031	0.000	-0.005
CXCL9	0.345*	0.090*	0.022	0.045	0.042	0.130*	0.081	-0.102*	-0.008	-0.073	-0.051	0.091*
DNER	-0.045	-0.085*	-0.031	-0.016	-0.189*	-0.085*	-0.363*	-0.100*	-0.202*	-0.145*	-0.196*	0.029
EN-RAGE	0.060	0.258*	0.182*	0.162*	0.040	0.111*	0.399*	0.249*	0.268*	-0.041	0.317*	0.086*
FGF-19	0.068	-0.056	-0.043	-0.061	-0.198*	0.053	-0.035	-0.016	-0.085	-0.057	0.106*	-0.014
FGF-21	0.219*	0.086*	0.118*	-0.044	0.259*	0.132*	-0.015	0.039	-0.059	0.159*	0.018	-0.106*
FGF-23	0.072	0.076	0.043	-0.026	0.163*	0.054	-0.040	-0.034	-0.100*	0.019	-0.089*	0.063
Flt3L	0.290*	0.051	-0.021	-0.100*	0.042	0.159*	-0.113*	-0.131*	-0.243*	0.043	-0.251*	0.079
GDNF	0.180*	0.070	0.115*	0.002	0.083*	0.114*	0.124*	0.091*	0.108*	-0.054	0.144*	0.024
HGF	0.335*	0.207*	0.275*	0.055	0.348*	0.027	0.271*	0.150*	0.182*	0.075	0.223*	-0.012
IFN-γ	-0.071	0.040	0.020	0.078	0.001	-0.024	0.107*	-0.091*	0.062	-0.001	-0.056	0.128*
IL-10	0.089*	0.064	0.082*	0.102*	-0.046	0.004	0.286*	0.079	0.312*	-0.037	0.151*	-0.057
IL-10RB	0.226*	0.117*	0.080	0.008	0.072	0.089*	0.119*	-0.005	-0.027	0.066	0.010	0.015
IL-12B	0.109*	0.073	-0.075	0.022	0.042	0.039	0.047	-0.103*	-0.121*	0.095*	-0.085*	0.106*
IL-18	0.199*	0.102*	0.165*	0.053	0.108*	0.012	0.096*	0.003	0.009	0.037	-0.009	-0.075
IL-18R1	0.173*	0.144*	0.130*	0.053	0.246*	-0.052	0.286*	0.171*	0.081	0.145*	0.162*	-0.022
IL-6	0.197*	0.485*	0.405*	0.212*	0.237*	0.120*	0.656*	0.163*	0.446*	0.021	0.379*	-0.062
IL-7	0.060	0.051	-0.014	0.022	0.080	-0.017	0.208*	0.114*	0.044	-0.039	0.073	0.042
IL-8	0.226*	0.081	0.061	-0.003	-0.004	0.130*	0.321*	0.050	0.156*	0.006	0.145*	-0.003
LIF-R	0.088*	-0.055	0.001	0.038	-0.051	0.013	-0.011	0.035	0.055	-0.024	0.024	-0.050
MCP-1	0.300*	0.040	0.100*	-0.036	0.047	0.200*	0.148*	-0.011	0.029	0.088*	-0.022	0.017
MCP-2	0.189*	0.124*	0.121*	0.063	0.088*	0.158*	0.021	-0.031	-0.051	0.116*	-0.032	0.080
MCP-3	0.178*	0.205*	0.143*	0.073	0.134*	0.202*	0.346*	0.107*	0.124*	0.116*	0.223*	-0.019
MCP-4	0.312*	0.047	0.041	-0.017	0.076	0.197*	-0.064	0.043	-0.142*	0.092*	-0.046	0.081
MMP-10	-0.017	0.103*	0.162*	0.009	-0.130*	-0.030	0.184*	0.053	-0.021	-0.166*	-0.032	-0.003
NT-3	-0.039	-0.016	-0.005	-0.017	-0.139*	-0.057	-0.030	-0.006	-0.083	0.056	0.023	-0.010
OPG	0.383*	0.123*	0.112*	0.031	0.059	0.270*	0.252*	0.098*	0.057	-0.061	0.110*	-0.011
OSM	0.074	0.131*	0.424*	0.220*	0.082	-0.062	0.301*	0.310*	0.355*	0.000	0.280*	-0.142*
SCF	0.018	-0.135*	-0.124*	-0.021	-0.176*	0.015	-0.361*	-0.198*	-0.103*	-0.032	-0.216*	-0.019
SIRT2	-0.016	-0.006	0.053	0.078	0.066	-0.077	0.187*	0.029	0.059	-0.040	0.103*	0.007
SLAMF1	0.146*	0.109*	0.118*	0.052	0.081	0.047	0.156*	0.074	0.013	0.095*	0.105*	0.015
STAMBP	0.010	-0.001	0.038	0.103*	0.053	-0.061	0.166*	0.026	0.059	-0.041	0.080	0.004
TGF-β1	0.246*	0.089*	0.079	0.036	0.052	0.009	0.226*	0.137*	-0.008	0.045	0.059	0.025
TNFB	-0.047	-0.093*	-0.078	-0.109*	-0.045	-0.126*	-0.223*	-0.146*	-0.257*	0.012	-0.127*	0.088*
TNFRSF9	0.193*	0.073	0.123*	-0.012	-0.010	0.029	0.027	-0.080	-0.165*	-0.004	-0.084*	0.057
TNFSF14	0.168*	0.269*	0.259*	0.076	0.277*	0.008	0.529*	0.286*	0.210*	0.099*	0.297*	-0.022
TRAIL	0.113*	-0.033	-0.044	0.001	0.055	-0.038	-0.395*	-0.057	-0.262*	0.089*	-0.284*	0.015
TRANCE	-0.029	-0.087*	-0.082*	-0.068	0.079	-0.068	-0.422*	-0.134*	-0.325*	0.161*	-0.288*	-0.016
TWEAK	0.054	-0.141*	-0.235*	-0.070	-0.152*	-0.049	-0.244*	-0.132*	-0.153*	-0.122*	-0.153*	0.062
uPA	0.075	-0.028	-0.029	-0.074	0.010	0.028	-0.043	-0.094*	-0.201*	0.044	-0.171*	0.059
VEGF-A	0.242*	0.238*	0.209*	0.052	0.175*	0.153*	0.390*	0.156*	0.133*	0.064	0.133*	-0.010

Age, at inclusion; high sensitivity CRP (hsCRP); white blood cell count (WBC); neutrophil-lymphocyte ratio (NLR); and body mass index (BMI). For cases, also acute stroke severity (NIH stroke scale, NIHSS); and number of days to blood sampling following the acute event. White, negligible ($-0.25 > r < 0.25$); blue, positive ($r > 0.25$); red, inverse ($r < -0.25$) correlations. *p<0.05

Table S4. Univariable regression analyses for each protein and all ischemic stroke during the acute-phase and at the 3-month and 7-year follow-up.

	Acute-phase (Univariable)		3-month follow-up (Univariable)		7-year follow-up (Univariable)	
Protein	OR (95% CI)	FDR, *q*	OR (95% CI)	FDR, *q*	OR (95% CI)	FDR, *q*
4E-BP1	1.11 (1.02-1.21)	2.51E-02	1.00 (0.91-1.09)	9.27E-01	0.99 (0.87-1.12)	8.73E-01
ADA	1.11 (0.86-1.42)	4.57E-01	1.22 (0.93-1.60)	1.70E-01	1.16 (0.81-1.65)	4.50E-01
AXIN1	2.59 (2.27-2.98)	1.60E-41	2.11 (1.85-2.41)	1.26E-27	1.47 (1.26-1.72)	2.22E-06
CASP-8	4.78 (3.62-6.38)	1.43E-26	3.68 (2.80-4.89)	4.00E-19	2.60 (1.84-3.71)	2.40E-07
CCL11	1.77 (1.39-2.25)	5.51E-06	1.92 (1.51-2.46)	3.15E-07	3.75 (2.68-5.31)	2.87E-13
CCL19	1.28 (1.12-1.47)	4.26E-04	1.60 (1.39-1.84)	1.98E-10	1.50 (1.26-1.78)	1.02E-05
CCL20	1.35 (1.20-1.53)	1.36E-06	1.41 (1.24-1.60)	2.64E-07	1.40 (1.21-1.62)	1.17E-05
CCL23	3.71 (2.81-4.94)	3.83E-19	1.96 (1.50-2.57)	1.78E-06	1.61 (1.12-2.32)	1.38E-02
CCL25	0.84 (0.70-1.01)	7.07E-02	1.24 (1.03-1.50)	2.96E-02	1.76 (1.37-2.26)	2.04E-05
CCL28	0.95 (0.72-1.25)	7.18E-01	0.66 (0.48-0.91)	1.62E-02	1.56 (1.04-2.37)	3.96E-02
CCL3	2.55 (2.00-3.27)	2.18E-13	1.56 (1.24-1.97)	2.50E-04	1.55 (1.18-2.07)	3.16E-03
CCL4	2.97 (2.37-3.74)	2.53E-20	1.78 (1.44-2.22)	3.89E-07	1.61 (1.24-2.10)	6.07E-04
CD244	1.91 (1.39-2.65)	1.24E-04	2.58 (1.85-3.62)	6.39E-08	2.34 (1.53-3.60)	1.64E-04
CD40	6.83 (4.93-9.62)	9.77E-29	4.37 (3.22-6.01)	1.23E-19	3.82 (2.62-5.65)	4.59E-11
CD5	1.40 (1.04-1.88)	3.29E-02	3.31 (2.40-4.60)	1.55E-12	2.83 (1.90-4.28)	1.18E-06
CD6	0.98 (0.79-1.22)	8.85E-01	1.58 (1.26-2.00)	1.65E-04	1.53 (1.15-2.06)	5.81E-03
CDCP1	1.80 (1.49-2.18)	2.18E-09	2.15 (1.77-2.64)	1.27E-13	1.97 (1.56-2.51)	6.43E-08
CSF-1	4.15 (2.87-6.07)	2.18E-13	2.91 (2.01-4.26)	6.39E-08	5.00 (3.08-8.22)	5.35E-10
CST5	0.63 (0.49-0.80)	2.94E-04	0.81 (0.63-1.02)	9.47E-02	1.41 (1.04-1.92)	3.63E-02
CX3CL1	1.00 (0.76-1.31)	9.96E-01	1.17 (0.89-1.56)	2.77E-01	2.43 (1.66-3.57)	1.07E-05
CXCL1	3.00 (2.65-3.43)	1.91E-60	2.26 (2.00-2.56)	1.66E-37	1.53 (1.33-1.77)	1.13E-08
CXCL10	1.33 (1.17-1.52)	3.67E-05	1.29 (1.13-1.47)	2.58E-04	1.69 (1.44-2.00)	1.55E-09
CXCL11	2.07 (1.79-2.41)	4.46E-21	1.91 (1.66-2.22)	2.08E-17	1.75 (1.46-2.10)	7.30E-09
CXCL5	2.15 (1.98-2.35)	8.13E-67	1.85 (1.71-2.00)	1.20E-50	1.40 (1.28-1.53)	2.87E-13
CXCL6	1.83 (1.54-2.19)	2.13E-11	1.15 (0.98-1.35)	1.06E-01	0.64 (0.49-0.82)	8.30E-04
CXCL9	1.91 (1.64-2.23)	6.90E-16	1.93 (1.64-2.27)	5.51E-15	2.45 (2.01-3.02)	1.26E-16
DNER	0.67 (0.46-0.96)	3.86E-02	0.55 (0.38-0.80)	2.26E-03	0.72 (0.44-1.18)	2.29E-01
EN-RAGE	3.15 (2.58-3.88)	1.51E-27	2.19 (1.82-2.66)	3.38E-15	1.96 (1.55-2.49)	6.43E-08
FGF-19	1.19 (1.05-1.35)	8.71E-03	1.19 (1.05-1.35)	8.53E-03	1.25 (1.06-1.48)	9.22E-03
FGF-21	1.23 (1.12-1.36)	3.21E-05	1.38 (1.25-1.52)	9.65E-10	1.30 (1.15-1.46)	4.18E-05
FGF-23	3.08 (2.37-4.04)	3.83E-16	2.59 (2.01-3.38)	2.32E-12	5.78 (4.01-8.53)	1.10E-18
Flt3L	0.33 (0.25-0.42)	2.64E-16	1.86 (1.43-2.43)	8.22E-06	4.27 (2.98-6.21)	7.41E-14
GDNF	2.69 (2.06-3.53)	1.69E-12	1.91 (1.47-2.49)	2.39E-06	2.76 (1.97-3.92)	2.20E-08
HGF	12.26 (8.87-17.26)	4.44E-48	6.35 (4.76-8.60)	2.33E-33	6.84 (4.76-10.03)	1.52E-22
IFN-γ	1.22 (0.98-1.53)	9.51E-02	1.28 (1.03-1.60)	3.64E-02	1.33 (1.02-1.75)	4.53E-02
IL-10	2.07 (1.66-2.61)	5.27E-10	1.50 (1.23-1.87)	2.06E-04	1.50 (1.18-1.94)	2.12E-03
IL-10RB	1.16 (0.89-1.51)	3.12E-01	1.97 (1.49-2.62)	5.49E-06	2.49 (1.73-3.63)	2.98E-06
IL-12B	1.12 (0.95-1.33)	2.09E-01	1.80 (1.50-2.16)	9.58E-10	1.73 (1.37-2.19)	1.10E-05
IL-18	1.35 (1.11-1.65)	4.58E-03	1.39 (1.14-1.71)	2.13E-03	1.48 (1.14-1.93)	4.84E-03
IL-18R1	4.09 (3.11-5.44)	2.15E-22	3.13 (2.38-4.16)	3.99E-15	3.10 (2.18-4.44)	1.66E-09
IL-6	2.82 (2.40-3.35)	8.01E-34	1.79 (1.55-2.09)	1.62E-13	1.82 (1.52-2.19)	5.35E-10
IL-7	2.99 (2.45-3.69)	4.24E-25	1.82 (1.52-2.20)	6.46E-10	1.13 (0.90-1.42)	3.22E-01
IL-8	1.74 (1.46-2.09)	3.60E-09	1.15 (0.97-1.36)	1.25E-01	1.25 (1.00-1.55)	5.72E-02
LIF-R	1.68 (1.21-2.34)	2.96E-03	1.42 (1.02-1.98)	4.48E-02	1.97 (1.31-2.98)	1.76E-03
MCP-1	1.29 (1.00-1.67)	6.41E-02	1.91 (1.45-2.54)	8.15E-06	4.03 (2.79-5.93)	2.40E-12
MCP-2	2.40 (1.97-2.94)	4.87E-17	2.46 (2.00-3.04)	2.33E-16	2.42 (1.86-3.17)	3.22E-10
MCP-3	4.11 (3.24-5.28)	5.01E-29	2.63 (2.10-3.34)	1.16E-15	2.27 (1.76-2.97)	2.12E-09
MCP-4	1.91 (1.56-2.34)	5.99E-10	1.61 (1.32-1.96)	5.41E-06	1.79 (1.39-2.33)	1.90E-05
MMP-10	1.04 (0.89-1.22)	6.57E-01	1.13 (0.96-1.33)	1.45E-01	1.53 (1.24-1.89)	1.41E-04
NT-3	1.19 (0.93-1.52)	1.91E-01	1.30 (1.01-1.69)	5.13E-02	0.97 (0.68-1.36)	8.90E-01
OPG	1.50 (1.12-2.02)	8.37E-03	2.44 (1.80-3.33)	2.89E-08	3.65 (2.45-5.48)	1.09E-09
OSM	5.62 (4.65-6.90)	1.70E-64	3.89 (3.28-4.65)	1.20E-50	3.67 (2.97-4.60)	1.04E-29
SCF	0.51 (0.40-0.65)	1.78E-07	0.80 (0.62-1.04)	1.06E-01	1.68 (1.18-2.43)	6.53E-03
SIRT2	1.38 (1.26-1.52)	2.58E-11	1.20 (1.09-1.32)	3.09E-04	0.83 (0.71-0.97)	2.40E-02
SLAMF1	1.39 (1.11-1.74)	5.48E-03	1.77 (1.40-2.25)	4.31E-06	2.42 (1.78-3.32)	6.12E-08
STAMPB	1.67 (1.46-1.90)	6.20E-14	1.43 (1.25-1.64)	3.17E-07	0.99 (0.82-1.21)	9.59E-01
TGF-β1	7.00 (5.18-9.61)	4.81E-34	5.98 (4.46-8.14)	2.49E-30	3.49 (2.50-4.95)	3.78E-12
TNFB	0.63 (0.50-0.80)	2.33E-04	1.04 (0.82-1.32)	7.47E-01	1.02 (0.73-1.42)	9.40E-01
TNFRSF9	1.57 (1.24-2.01)	3.96E-04	3.03 (2.33-3.98)	1.91E-15	2.63 (1.91-3.66)	1.53E-08
TNFSF14	11.86 (8.60-16.63)	6.57E-48	6.08 (4.53-8.27)	7.62E-31	4.21 (2.96-6.08)	4.93E-14
TRAIL	1.28 (0.95-1.72)	1.15E-01	1.28 (0.94-1.76)	1.38E-01	1.52 (1.01-2.30)	5.40E-02
TRANCE	1.45 (1.22-1.74)	7.18E-05	1.23 (1.02-1.48)	4.13E-02	1.10 (0.85-1.41)	5.09E-01
TWEAK	0.79 (0.60-1.03)	9.62E-02	1.16 (0.86-1.58)	3.45E-01	1.21 (0.80-1.84)	3.99E-01
uPA	0.25 (0.17-0.35)	1.90E-13	0.81 (0.57-1.14)	2.38E-01	1.02 (0.63-1.62)	9.59E-01
VEGF-A	7.59 (5.40-10.84)	2.64E-29	5.97 (4.28-8.43)	3.26E-24	7.10 (4.70-10.92)	1.10E-18

White, non significant ($q > 0.05$); blue, significant ($q < 0.05$) and elevated in cases; red, significant ($q < 0.05$) and lower in cases compared to controls.

Table S5. Multivariable regression analyses using a two-step random-split approach to simulate a discovery and validation cohort for the acute and 3-month follow-up.

	Acute-phase (Multivariable)				3-month follow-up (Multivariable)			
	Random sample 1		Random sample 2		Random sample 1		Random sample 2	
Protein	OR (95% CI)	FDR, *q*	OR (95% CI)	FDR, *q*	OR (95% CI)	FDR, *q*	OR (95% CI)	FDR, *q*
4E-BP1	1.2 (1.1-1.4)	1.63E-02	1.0 (0.9-1.1)	8.45E-01	1.1 (0.9-1.2)	4.46E-01	0.9 (0.8-1.0)	2.19E-01
ADA	1.2 (0.8-1.8)	5.07E-01	0.9 (0.6-1.4)	7.31E-01	1.2 (0.8-1.9)	4.42E-01	1.1 (0.7-1.7)	7.32E-01
AXIN1	2.9 (2.3-3.6)	1.63E-19	2.5 (2.0-3.0)	1.52E-17	2.4 (1.9-2.9)	1.43E-13	1.9 (1.6-2.3)	2.84E-09
CASP-8	5.6 (3.6-8.8)	9.45E-13	4.4 (2.8-6.8)	1.18E-09	3.4 (2.2-5.2)	6.75E-07	4.6 (2.9-7.1)	2.35E-09
CCL11	1.5 (1-2.3)	8.62E-02	1.6 (1.1-2.3)	7.76E-02	1.9 (1.2-2.9)	1.08E-02	1.7 (1.2-2.5)	1.98E-02
CCL19	1.3 (1.0-1.6)	4.08E-02	1.1 (0.9-1.4)	4.74E-01	1.6 (1.2-1.9)	8.41E-04	1.6 (1.2-1.9)	5.84E-04
CCL20	1.5 (1.2-1.8)	5.48E-04	1.1 (0.9-1.3)	6.06E-01	1.5 (1.2-1.9)	5.29E-04	1.2 (1.0-1.4)	1.55E-01
CCL23	3.2 (2.2-4.8)	1.15E-07	4.6 (2.8-7.4)	1.38E-08	1.8 (1.2-2.7)	2.06E-02	2.3 (1.5-3.6)	5.46E-04
CCL25	0.6 (0.5-0.9)	1.03E-02	0.9 (0.6-1.2)	5.35E-01	1.0 (0.8-1.4)	8.83E-01	1.3 (1.0-1.8)	1.42E-01
CCL28	0.9 (0.6-1.4)	7.37E-01	0.9 (0.6-1.4)	6.59E-01	0.5 (0.3-0.9)	3.65E-02	0.7 (0.5-1.2)	2.92E-01
CCL3	3.4 (2.3-5.2)	6.83E-08	1.6 (1.1-2.3)	2.29E-02	1.7 (1.2-2.5)	2.06E-02	1.2 (0.9-1.7)	3.48E-01
CCL4	3.7 (2.5-5.3)	4.20E-11	2.2 (1.6-3.1)	6.23E-05	2.0 (1.4-2.9)	5.24E-04	1.5 (1.0-2.1)	6.30E-02
CD244	2.1 (1.2-3.5)	1.63E-02	2.1 (1.3-3.5)	1.58E-02	2.8 (1.6-4.7)	1.11E-03	3.0 (1.8-5.1)	2.56E-04
CD40	7.7 (4.6-13.0)	4.79E-13	7.8 (4.6-13.5)	7.06E-12	5.2 (3.1-8.6)	8.09E-09	3.9 (2.4-6.3)	7.98E-07
CD5	1.1 (0.7-1.8)	6.54E-01	1.3 (0.8-2.0)	5.55E-01	2.8 (1.7-4.5)	5.29E-04	3.7 (2.2-6.2)	1.63E-05
CD6	0.9 (0.7-1.3)	7.37E-01	1.1 (0.8-1.6)	6.68E-01	1.6 (1.1-2.2)	2.99E-02	1.8 (1.2-2.6)	8.29E-03
CDCP1	2.0 (1.5-2.8)	7.55E-05	1.4 (1.0-1.9)	1.54E-01	2.5 (1.8-3.5)	2.48E-06	1.9 (1.3-2.6)	1.86E-03
CSF-1	4.9 (2.8-8.8)	4.68E-07	3.0 (1.7-5.5)	2.41E-03	2.8 (1.5-5.1)	3.89E-03	2.8 (1.5-5.0)	3.29E-03
CST5	0.7 (0.5-1.0)	7.68E-02	0.8 (0.6-1.3)	6.06E-01	0.9 (0.6-1.3)	7.35E-01	1.2 (0.8-1.8)	5.22E-01
CX3CL1	0.9 (0.6-1.5)	7.74E-01	0.9 (0.6-1.4)	7.44E-01	1.1 (0.7-1.7)	8.12E-01	1.2 (0.8-1.9)	5.10E-01
CXCL1	3.1 (2.5-3.7)	5.20E-27	3.1 (2.5-3.8)	9.97E-24	2.3 (1.9-2.7)	3.01E-15	2.2 (1.9-2.7)	1.68E-14
CXCL10	1.4 (1.1-1.7)	9.03E-03	1.4 (1.2-1.8)	4.64E-03	1.3 (1.1-1.6)	2.99E-02	1.4 (1.2-1.7)	3.61E-03
CXCL11	2.1 (1.7-2.7)	2.49E-09	2.2 (1.7-2.7)	1.18E-09	1.9 (1.5-2.4)	4.33E-07	2.1 (1.6-2.6)	8.95E-09
CXCL5	2.3 (2.0-2.6)	3.71E-29	2.1 (1.9-2.4)	3.64E-25	2.0 (1.7-2.2)	7.87E-22	1.8 (1.6-2.0)	3.06E-18
CXCL6	1.5 (1.2-2.0)	5.57E-03	2.2 (1.6-2.9)	1.27E-06	0.9 (0.7-1.2)	5.21E-01	1.3 (1.0-1.7)	9.80E-02
CXCL9	2.3 (1.7-2.9)	1.99E-08	2.0 (1.6-2.6)	5.06E-07	2.4 (1.8-3.3)	8.89E-08	2.1 (1.6-2.7)	3.28E-07
DNER	0.6 (0.4-1.1)	1.81E-01	0.6 (0.3-1.1)	2.81E-01	0.5 (0.3-0.9)	6.45E-02	0.5 (0.3-0.9)	3.53E-02
EN-RAGE	3.0 (2.3-4.1)	1.63E-12	2.8 (2.0-3.9)	1.25E-08	1.8 (1.4-2.4)	1.41E-04	2.2 (1.6-3.0)	4.36E-06
FGF-19	1.2 (1.0-1.5)	1.58E-01	1.2 (1.0-1.5)	1.83E-01	1.1 (0.9-1.4)	2.68E-01	1.3 (1.1-1.6)	3.53E-02
FGF-21	1.1 (1.0-1.3)	1.81E-01	1.1 (0.9-1.2)	6.58E-01	1.2 (1.1-1.5)	2.12E-02	1.3 (1.1-1.5)	1.27E-02
FGF-23	3.1 (2.0-4.7)	1.15E-06	2.7 (1.8-4.0)	8.27E-06	2.3 (1.6-3.5)	3.52E-04	2.5 (1.7-3.8)	1.23E-04
Flt3L	0.2 (0.1-0.3)	1.54E-10	0.4 (0.2-0.6)	1.72E-05	1.6 (1.0-2.4)	7.30E-02	2.3 (1.5-3.5)	1.08E-03
GDNF	2.9 (1.9-4.5)	4.33E-06	1.9 (1.2-2.9)	1.42E-02	2.2 (1.4-3.3)	2.70E-03	1.3 (0.9-2.0)	2.76E-01
HGF	15.8 (9.1-27.4)	2.34E-20	13.5 (7.7-23.6)	1.43E-17	8.2 (4.9-13.6)	1.43E-13	6.3 (3.9-10.1)	1.09E-11
IFN-γ	1.3 (0.9-1.8)	2.27E-01	1.2 (0.8-1.7)	5.35E-01	1.1 (0.8-1.5)	7.35E-01	1.5 (1.1-2.1)	4.03E-02
IL-10	2.2 (1.5-3.1)	7.55E-05	1.7 (1.2-2.3)	6.29E-03	1.3 (1.0-1.8)	1.57E-01	1.6 (1.1-2.2)	1.89E-02
IL-10RB	1.4 (0.9-2.0)	2.15E-01	0.7 (0.5-1.2)	4.04E-01	2.0 (1.3-3.1)	7.22E-03	1.6 (1.0-2.5)	9.80E-02
IL-12B	1.4 (1.1-1.9)	3.25E-02	1.3 (1.0-1.7)	1.54E-01	2.4 (1.7-3.3)	1.25E-06	2.3 (1.7-3.1)	7.98E-07
IL-18	1.2 (0.9-1.7)	2.29E-01	1.1 (0.8-1.5)	6.87E-01	1.2 (0.9-1.6)	4.42E-01	1.2 (0.9-1.7)	3.83E-01
IL-18R1	3.3 (2.2-5.2)	4.68E-07	4.0 (2.6-6.3)	1.38E-08	2.2 (1.4-3.4)	2.70E-03	3.3 (2.1-5.1)	1.97E-06
IL-6	2.5 (2.0-3.2)	5.92E-13	2.7 (2.0-3.5)	2.09E-11	1.5 (1.2-1.8)	4.14E-03	1.8 (1.4-2.3)	4.16E-05
IL-7	3.4 (2.4-4.6)	2.80E-12	2.7 (2.0-3.7)	3.05E-09	1.8 (1.3-2.4)	1.42E-03	1.8 (1.4-2.3)	2.37E-04
IL-8	1.5 (1.1-2.0)	1.03E-02	1.6 (1.2-2.1)	6.29E-03	0.8 (0.6-1.1)	3.51E-01	1.2 (0.9-1.5)	3.30E-01
LIF-R	1.5 (0.9-2.5)	2.27E-01	1.9 (1.2-3.3)	4.27E-02	1.4 (0.8-2.3)	3.76E-01	1.8 (1.1-3.1)	5.56E-02
MCP-1	1.3 (0.9-2.0)	2.62E-01	1.0 (0.6-1.5)	8.48E-01	2.3 (1.4-3.7)	4.14E-03	1.6 (1.1-2.4)	4.03E-02
MCP-2	2.5 (1.8-3.5)	1.42E-07	2.4 (1.7-3.2)	1.27E-06	2.6 (1.9-3.6)	4.33E-07	2.5 (1.8-3.5)	6.98E-07
MCP-3	4.2 (2.9-6.0)	5.92E-13	3.3 (2.2-4.9)	3.52E-08	2.3 (1.7-3.3)	2.19E-05	2.5 (1.8-3.7)	9.20E-06
MCP-4	2.2 (1.6-3.0)	2.69E-05	1.6 (1.2-2.2)	6.29E-03	1.8 (1.3-2.5)	4.14E-03	1.5 (1.1-2.0)	2.62E-02
MMP-10	1.0 (0.8-1.3)	8.47E-01	0.8 (0.6-1.1)	2.58E-01	1.0 (0.8-1.3)	9.25E-01	1.0 (0.8-1.3)	7.77E-01
NT-3	1.4 (0.9-2.1)	2.28E-01	1.1 (0.8-1.6)	6.06E-01	1.5 (0.9-2.3)	1.55E-01	1.2 (0.8-1.6)	5.10E-01
OPG	1.2 (0.8-1.9)	5.50E-01	1.2 (0.7-2.1)	6.06E-01	2.0 (1.2-3.3)	1.64E-02	2.8 (1.6-4.7)	1.23E-03
OSM	7.3 (5.2-10.2)	1.85E-27	4.4 (3.4-5.8)	5.85E-24	4.1 (3.1-5.3)	2.74E-21	3.6 (2.8-4.7)	1.82E-18
SCF	0.7 (0.5-1.0)	8.62E-02	0.7 (0.4-1.0)	1.13E-01	1.2 (0.8-1.9)	4.55E-01	0.9 (0.6-1.4)	7.32E-01
SIRT2	1.5 (1.3-1.8)	4.68E-07	1.3 (1.1-1.5)	2.41E-03	1.3 (1.1-1.5)	6.03E-03	1.1 (1.0-1.3)	2.76E-01
SLAMF1	1.3 (0.9-1.9)	1.81E-01	1.2 (0.8-1.7)	5.66E-01	1.5 (1.0-2.2)	8.63E-02	1.8 (1.2-2.5)	7.99E-03
STAMBP	1.9 (1.5-2.3)	6.50E-08	1.6 (1.3-1.9)	9.62E-05	1.6 (1.3-1.9)	3.54E-04	1.3 (1.1-1.6)	3.53E-02
TGF-β1	7.6 (4.7-12.2)	5.29E-15	5.8 (3.6-9.4)	1.46E-11	5.5 (3.5-8.7)	2.02E-11	5.9 (3.7-9.4)	1.58E-11
TNFB	0.6 (0.4-0.9)	2.54E-02	0.7 (0.5-1.0)	1.11E-01	1.1 (0.7-1.6)	7.35E-01	1.2 (0.8-1.7)	3.88E-01
TNFRSF9	1.9 (1.3-2.8)	1.91E-03	1.2 (0.8-1.8)	5.66E-01	4.4 (2.8-7.1)	1.31E-08	2.2 (1.5-3.3)	3.49E-04
TNFSF14	13.5 (8.1-22.4)	5.69E-21	10.9 (6.5-18.2)	1.43E-17	5.7 (3.6-9.1)	3.00E-11	5.8 (3.6-9.3)	1.60E-11
TRAIL	1.1 (0.7-1.8)	6.54E-01	1.2 (0.7-1.9)	6.59E-01	1.3 (0.8-2.1)	4.46E-01	1.1 (0.7-1.8)	7.56E-01
TRANCE	1.7 (1.3-2.2)	6.11E-04	1.4 (1.0-1.8)	9.14E-02	1.5 (1.1-2.1)	1.26E-02	1.1 (0.8-1.4)	7.56E-01
TWEAK	0.9 (0.6-1.3)	6.25E-01	1.3 (0.8-2.1)	4.74E-01	1.6 (1.0-2.6)	1.40E-01	1.9 (1.2-3.2)	3.19E-02
uPA	0.3 (0.1-0.5)	1.46E-05	0.2 (0.1-0.4)	1.70E-06	0.8 (0.5-1.4)	6.55E-01	0.9 (0.5-1.5)	6.80E-01
VEGF-A	7.1 (4.2-11.9)	3.39E-12	6.7 (3.8-12.0)	2.76E-09	5.5 (3.2-9.4)	1.59E-08	5.7 (3.3-9.7)	9.79E-09

White, non significant (q > 0.05); blue, significant (q < 0.05) and elevated in cases; red, significant (q < 0.05) and lower in cases compared to controls.

Table S6. Multivariable regression analyses from sensitivity analyses to exclude either cases with clinical signs of infection at the time of blood drawn (acute-phase) or cases that experienced a recurrent stroke or another vascular event during follow-up.

	Acute-phase n=18 infections excluded		3-month follow-up n=32 vascular events excluded		7-year follow-up n=47 vascular events excluded	
Protein	OR (95% CI)	FDR, *q*	OR (95% CI)	FDR, *q*	OR (95% CI)	FDR, *q*
4E-BP1	1.1 (1.0-1.2)	0.117	1.0 (0.9-1.1)	0.987	1.0 (0.8-1.1)	0.580
ADA	1.0 (0.7-1.3)	0.995	1.2 (0.9-1.6)	0.346	1.3 (0.8-2.0)	0.370
AXIN1	2.6 (2.3-3.0)	<0.001	2.1 (1.8-2.4)	<0.001	1.3 (1.1-1.6)	0.006
CASP-8	4.8 (3.5-6.6)	<0.001	4.0 (2.9-5.5)	<0.001	2.4 (1.5-3.7)	<0.001
CCL11	1.6 (1.2-2.1)	<0.001	1.8 (1.4-2.4)	<0.001	3.3 (2.1-5.4)	<0.001
CCL19	1.2 (1.1-1.4)	0.010	1.5 (1.3-1.8)	<0.001	1.2 (1.0-1.6)	0.107
CCL20	1.2 (1.1-1.4)	0.003	1.3 (1.1-1.5)	<0.001	1.5 (1.2-1.8)	<0.001
CCL23	3.5 (2.6-4.8)	<0.001	2.1 (1.5-2.8)	<0.001	1.6 (1.0-2.5)	0.088
CCL25	0.7 (0.6-0.9)	0.009	1.1 (0.9-1.4)	0.280	1.6 (1.2-2.2)	0.009
CCL28	0.9 (0.7-1.3)	0.648	0.6 (0.4-0.9)	0.020	1.7 (1.0-2.9)	0.047
CCL3	2.3 (1.7-3.0)	<0.001	1.4 (1.1-1.8)	0.011	1.4 (1.0-2.0)	0.088
CCL4	2.8 (2.2-3.6)	<0.001	1.7 (1.4-2.2)	<0.001	1.6 (1.1-2.2)	0.011
CD244	2.2 (1.5-3.2)	<0.001	2.9 (2.0-4.1)	<0.001	3.2 (1.8-5.6)	<0.001
CD40	7.3 (5.0-10.5)	<0.001	4.4 (3.1-6.3)	<0.001	3.6 (2.2-5.9)	<0.001
CD5	1.2 (0.9-1.7)	0.217	3.1 (2.1-4.4)	<0.001	2.5 (1.5-4.1)	<0.001
CD6	1.1 (0.8-1.4)	0.648	1.7 (1.3-2.1)	<0.001	1.7 (1.2-2.4)	0.012
CDCP1	1.7 (1.4-2.2)	<0.001	2.2 (1.7-2.8)	<0.001	1.9 (1.3-2.6)	<0.001
CSF-1	3.6 (2.4-5.4)	<0.001	2.7 (1.8-4.2)	<0.001	3.9 (2.1-7.3)	<0.001
CST5	0.8 (0.6-1.0)	0.062	1.0 (0.8-1.3)	0.930	1.8 (1.2-2.8)	0.009
CX3CL1	0.9 (0.7-1.3)	0.639	1.1 (0.8-1.5)	0.503	2.6 (1.5-4.3)	<0.001
CXCL1	3.0 (2.6-3.5)	<0.001	2.2 (2.0-2.5)	<0.001	1.5 (1.3-1.8)	<0.001
CXCL10	1.4 (1.2-1.6)	<0.001	1.4 (1.2-1.6)	<0.001	1.6 (1.3-1.9)	<0.001
CXCL11	2.1 (1.8-2.5)	<0.001	2.0 (1.7-2.4)	<0.001	1.8 (1.4-2.2)	<0.001
CXCL5	2.2 (2.0-2.4)	<0.001	1.9 (1.7-2.0)	<0.001	1.4 (1.2-1.5)	<0.001
CXCL6	1.8 (1.5-2.2)	<0.001	1.1 (0.9-1.3)	0.330	0.8 (0.6-1.0)	0.101
CXCL9	2.1 (1.8-2.6)	<0.001	2.2 (1.8-2.7)	<0.001	2.3 (1.8-3.0)	<0.001
DNER	0.7 (0.4-1.0)	0.092	0.5 (0.3-0.8)	<0.001	1.0 (0.5-1.9)	0.926
EN-RAGE	2.8 (2.3-3.5)	<0.001	2.0 (1.6-2.4)	<0.001	1.6 (1.2-2.1)	<0.001
FGF-19	1.2 (1.1-1.4)	0.005	1.2 (1.0-1.4)	0.018	1.3 (1.0-1.6)	0.043
FGF-21	1.1 (1.0-1.2)	0.153	1.2 (1.1-1.4)	<0.001	1.2 (1.0-1.4)	0.038
FGF-23	3.0 (2.2-4.0)	<0.001	2.4 (1.8-3.2)	<0.001	4.0 (2.6-6.1)	<0.001
Flt3L	0.3 (0.2-0.4)	<0.001	2.0 (1.4-2.7)	<0.001	3.4 (2.1-5.5)	<0.001
GDNF	2.3 (1.7-3.2)	<0.001	1.8 (1.3-2.4)	<0.001	2.5 (1.6-3.9)	<0.001
HGF	13.7 (9.3-20.3)	<0.001	7.0 (5.0-9.9)	<0.001	6.7 (4.0-11.2)	<0.001
IFN-γ	1.2 (1.0-1.6)	0.117	1.3 (1.0-1.6)	0.046	1.3 (0.9-1.8)	0.144
IL-10	1.8 (1.4-2.3)	<0.001	1.4 (1.2-1.8)	<0.001	1.4 (1.1-1.9)	0.035
IL-10RB	1.0 (0.8-1.4)	0.810	1.8 (1.3-2.5)	<0.001	1.9 (1.2-3.0)	0.012
IL-12B	1.4 (1.2-1.7)	<0.001	2.3 (1.9-2.9)	<0.001	1.8 (1.3-2.5)	<0.001
IL-18	1.2 (1.0-1.5)	0.153	1.2 (1.0-1.6)	0.081	1.4 (1.0-2.0)	0.086
IL-18R1	3.5 (2.6-4.8)	<0.001	2.7 (2.0-3.7)	<0.001	2.9 (1.8-4.6)	<0.001
IL-6	2.5 (2.1-3.0)	<0.001	1.6 (1.4-1.9)	<0.001	1.6 (1.3-2.1)	<0.001
IL-7	2.9 (2.4-3.7)	<0.001	1.8 (1.5-2.2)	<0.001	1.2 (0.9-1.5)	0.370
IL-8	1.5 (1.2-1.8)	<0.001	1.0 (0.8-1.2)	0.916	1.1 (0.8-1.5)	0.610
LIF-R	1.7 (1.2-2.5)	0.008	1.6 (1.1-2.3)	0.014	2.0 (1.2-3.5)	0.015
MCP-1	1.1 (0.8-1.5)	0.607	1.8 (1.3-2.5)	<0.001	3.9 (2.4-6.4)	<0.001
MCP-2	2.5 (2.0-3.1)	<0.001	2.5 (2.0-3.2)	<0.001	2.4 (1.7-3.3)	<0.001
MCP-3	3.7 (2.8-4.8)	<0.001	2.4 (1.9-3.1)	<0.001	1.8 (1.3-2.5)	<0.001
MCP-4	2.0 (1.6-2.5)	<0.001	1.6 (1.3-2.0)	<0.001	1.5 (1.1-2.1)	0.037
MMP-10	0.9 (0.7-1.0)	0.135	1.0 (0.8-1.2)	0.901	1.5 (1.1-2.0)	0.008
NT-3	1.3 (1.0-1.7)	0.130	1.3 (1.0-1.7)	0.101	1.0 (0.7-1.6)	0.895
OPG	1.1 (0.8-1.6)	0.628	2.3 (1.6-3.3)	<0.001	3.0 (1.7-5.2)	<0.001
OSM	5.5 (4.4-6.8)	<0.001	3.9 (3.2-4.7)	<0.001	3.9 (2.9-5.1)	<0.001
SCF	0.7 (0.5-1.0)	0.033	1.0 (0.8-1.4)	0.899	1.5 (1.0-2.5)	0.097
SIRT2	1.4 (1.3-1.5)	<0.001	1.2 (1.1-1.4)	<0.001	0.8 (0.7-1.0)	0.038
SLAMF1	1.2 (1.0-1.6)	0.117	1.7 (1.3-2.2)	<0.001	2.1 (1.4-3.1)	<0.001
STAMBP	1.7 (1.5-2.0)	<0.001	1.5 (1.3-1.7)	<0.001	0.9 (0.7-1.2)	0.577
TGF-β1	6.4 (4.6-8.9)	<0.001	5.7 (4.1-8.0)	<0.001	3.3 (2.2-5.1)	<0.001
TNFB	0.7 (0.5-0.9)	0.008	1.2 (0.9-1.5)	0.287	1.1 (0.7-1.7)	0.751
TNFRSF9	1.6 (1.2-2.0)	<0.001	2.9 (2.2-4.0)	<0.001	2.2 (1.4-3.3)	<0.001
TNFSF14	11.4 (8.0-16.4)	<0.001	5.7 (4.1-7.9)	<0.001	3.9 (2.4-6.1)	<0.001
TRAIL	1.3 (0.9-1.8)	0.153	1.2 (0.8-1.7)	0.346	1.6 (1.0-2.7)	0.092
TRANCE	1.7 (1.4-2.1)	<0.001	1.3 (1.0-1.6)	0.021	1.2 (0.9-1.7)	0.244
TWEAK	1.1 (0.8-1.5)	0.607	1.7 (1.2-2.4)	0.004	1.3 (0.8-2.3)	0.342
uPA	0.2 (0.2-0.4)	<0.001	0.8 (0.6-1.2)	0.433	1.6 (0.8-2.9)	0.180
VEGF-A	6.4 (4.4-9.4)	<0.001	5.7 (3.9-8.3)	<0.001	5.8 (3.4-10.0)	<0.001

White, non significant ($q > 0.05$); blue, significant ($q < 0.05$) and elevated in cases; red, significant ($q < 0.05$) and lower in cases compared to controls.

Table S7. Multivariable regression analyses using just the subgroup of 223 cases who participated in the 7-year follow-up.

	Acute-phase (Multivariable)		3-month follow-up (Multivariable)	
Protein	OR (95% CI)	FDR, *q*	OR (95% CI)	FDR, *q*
4E-BP1	1.10 (0.99-1.21)	8.59E-02	0.99 (0.89-1.10)	8.72E-01
ADA	1.05 (0.79-1.40)	7.59E-01	1.17 (0.87-1.58)	3.38E-01
AXIN1	2.63 (2.28-3.04)	9.98E-39	2.11 (1.83-2.42)	1.12E-24
CASP-8	4.99 (3.66-6.82)	2.56E-23	3.96 (2.91-5.39)	1.28E-17
CCL11	1.53 (1.15-2.02)	4.82E-03	1.77 (1.33-2.35)	1.43E-04
CCL19	1.22 (1.05-1.41)	1.31E-02	1.55 (1.33-1.81)	6.43E-08
CCL20	1.24 (1.09-1.41)	1.72E-03	1.31 (1.14-1.49)	1.43E-04
CCL23	3.72 (2.73-5.05)	2.26E-16	2.01 (1.50-2.70)	7.14E-06
CCL25	0.73 (0.59-0.90)	4.30E-03	1.13 (0.92-1.39)	2.90E-01
CCL28	0.88 (0.65-1.20)	4.51E-01	0.62 (0.43-0.89)	1.22E-02
CCL3	2.31 (1.76-3.03)	3.71E-09	1.41 (1.10-1.82)	1.06E-02
CCL4	2.83 (2.21-3.63)	4.69E-16	1.72 (1.35-2.19)	2.01E-05
CD244	2.08 (1.45-2.99)	1.36E-04	2.88 (1.99-4.16)	5.17E-08
CD40	7.49 (5.18-10.81)	6.00E-26	4.38 (3.11-6.19)	2.19E-16
CD5	1.18 (0.85-1.64)	3.58E-01	3.05 (2.14-4.36)	2.71E-09
CD6	1.00 (0.79-1.28)	9.89E-01	1.65 (1.28-2.12)	1.78E-04
CDCP1	1.70 (1.35-2.13)	1.13E-05	2.18 (1.71-2.77)	9.16E-10
CSF-1	3.86 (2.57-5.81)	2.49E-10	2.73 (1.80-4.14)	5.27E-06
CST5	0.74 (0.56-0.97)	3.94E-02	1.01 (0.77-1.32)	9.39E-01
CX3CL1	0.94 (0.69-1.27)	7.13E-01	1.15 (0.84-1.57)	4.29E-01
CXCL1	3.05 (2.65-3.51)	3.01E-53	2.24 (1.97-2.55)	2.82E-32
CXCL10	1.39 (1.21-1.61)	1.40E-05	1.36 (1.18-1.58)	5.89E-05
CXCL11	2.16 (1.84-2.55)	7.86E-20	2.01 (1.71-2.35)	7.04E-17
CXCL5	2.18 (1.99-2.40)	7.56E-59	1.86 (1.71-2.03)	4.87E-44
CXCL6	1.82 (1.50-2.20)	2.78E-09	1.10 (0.92-1.31)	3.59E-01
CXCL9	2.11 (1.76-2.52)	1.18E-15	2.22 (1.84-2.68)	9.74E-16
DNER	0.62 (0.41-0.94)	3.37E-02	0.50 (0.34-0.76)	1.57E-03
EN-RAGE	2.89 (2.34-3.58)	8.18E-22	1.99 (1.62-2.43)	7.92E-11
FGF-19	1.19 (1.04-1.37)	1.73E-02	1.20 (1.05-1.38)	1.15E-02
FGF-21	1.10 (0.98-1.22)	1.23E-01	1.25 (1.12-1.40)	1.09E-04
FGF-23	2.81 (2.11-3.74)	5.11E-12	2.39 (1.80-3.17)	4.08E-09
Flt3L	0.28 (0.20-0.37)	4.46E-16	1.91 (1.41-2.58)	5.57E-05
GDNF	2.41 (1.79-3.25)	1.89E-08	1.70 (1.27-2.28)	6.60E-04
HGF	14.19 (9.62-20.91)	8.14E-40	6.86 (4.87-9.66)	4.19E-27
IFN-γ	1.24 (0.97-1.59)	1.05E-01	1.28 (1.01-1.62)	5.24E-02
IL-10	1.92 (1.51-2.44)	1.91E-07	1.44 (1.15-1.80)	2.09E-03
IL-10RB	1.03 (0.76-1.38)	8.71E-01	1.76 (1.29-2.39)	5.47E-04
IL-12B	1.35 (1.11-1.64)	4.61E-03	2.31 (1.87-2.87)	9.79E-14
IL-18	1.19 (0.95-1.49)	1.57E-01	1.22 (0.97-1.53)	1.08E-01
IL-18R1	3.64 (2.68-4.96)	7.78E-16	2.65 (1.95-3.60)	1.49E-09
IL-6	2.55 (2.14-3.03)	3.41E-25	1.61 (1.36-1.90)	5.68E-08
IL-7	3.03 (2.43-3.78)	3.04E-22	1.76 (1.45-2.15)	5.17E-08
IL-8	1.55 (1.28-1.88)	1.44E-05	1.02 (0.85-1.23)	8.78E-01
LIF-R	1.68 (1.17-2.42)	7.70E-03	1.54 (1.07-2.21)	2.63E-02
MCP-1	1.13 (0.85-1.51)	4.51E-01	1.84 (1.36-2.50)	1.65E-04
MCP-2	2.41 (1.93-3.02)	4.35E-14	2.54 (2.01-3.19)	1.45E-14
MCP-3	3.77 (2.89-4.91)	4.00E-22	2.45 (1.91-3.16)	1.05E-11
MCP-4	1.87 (1.50-2.33)	7.52E-08	1.60 (1.28-2.00)	5.65E-05
MMP-10	0.89 (0.75-1.07)	2.45E-01	1.01 (0.84-1.21)	9.32E-01
NT-3	1.22 (0.93-1.60)	1.78E-01	1.27 (0.97-1.68)	1.03E-01
OPG	1.20 (0.85-1.70)	3.39E-01	2.26 (1.58-3.24)	1.94E-05
OSM	5.56 (4.50-6.87)	9.62E-56	3.82 (3.17-4.61)	9.74E-44
SCF	0.67 (0.51-0.88)	6.77E-03	1.06 (0.79-1.42)	7.39E-01
SIRT2	1.40 (1.26-1.56)	3.99E-10	1.20 (1.08-1.33)	1.18E-03
SLAMF1	1.23 (0.96-1.58)	1.23E-01	1.61 (1.24-2.10)	5.47E-04
STAMPB	1.71 (1.48-1.97)	9.51E-13	1.44 (1.24-1.66)	2.68E-06
TGF-β1	6.57 (4.72-9.14)	7.34E-28	5.67 (4.10-7.83)	8.55E-25
TNFB	0.65 (0.50-0.84)	1.86E-03	1.17 (0.90-1.52)	2.90E-01
TNFRSF9	1.51 (1.17-1.97)	3.19E-03	2.99 (2.23-4.01)	8.98E-13
TNFSF14	11.86 (8.29-16.97)	1.42E-40	5.65 (4.07-7.83)	2.78E-24
TRAIL	1.15 (0.83-1.59)	4.51E-01	1.19 (0.84-1.67)	3.72E-01
TRANCE	1.54 (1.26-1.88)	4.25E-05	1.28 (1.04-1.57)	2.35E-02
TWEAK	1.04 (0.76-1.41)	8.44E-01	1.74 (1.23-2.47)	2.72E-03
uPA	0.23 (0.16-0.35)	5.79E-12	0.85 (0.58-1.22)	4.13E-01
VEGF-A	6.78 (4.63-9.92)	3.66E-22	5.46 (3.76-7.91)	2.96E-18

White, non significant ($q > 0.05$); blue, significant ($q < 0.05$) and elevated in cases; red, significant ($q < 0.05$) and lower in cases compared to controls.
